# Traceability and Health Risk Assessment in the Feed–Animal–Milk Chain: A Comparative Evaluation of Traditional and Industrial Dairy Systems in Romania

**DOI:** 10.3390/foods15162844

**Published:** 2026-08-14

**Authors:** Ionela Ramona Zgavarogea, Nadia Paun, Claudia Sandru, Violeta-Carolina Niculescu, Ana Maria Nasture, Marius-Gheorghe Miricioiu

**Affiliations:** 1 National Research and Development Institute for Cryogenic and Isotopic Technologies—ICSI Ramnicu Vâlcea, 4th Uzinei Street, P.O. Box Raureni 7, 240050 Ramnicu Valcea, Romania; ramona.zgavarogea@icsi.ro (I.R.Z.); ana.nasture@icsi.ro (A.M.N.);; 2 DAIC SYSTEM S.R.L., 58 Fantanii Street, 240301 Ramnicu Valcea, Romania

**Keywords:** dairy processing, environmental exposure, food authenticity, geochemical fingerprinting, ICP-OES, regional traceability

## Abstract

Cow milk and dairy products may reflect environmental elemental inputs transferred through the feed–animal–milk chain. This study investigated the elemental traceability, regional differentiation, dairy processing effects, and dietary exposure associated with milk and dairy products from two contrasting Romanian dairy production systems: the traditional Vaideeni region and the industrial Ibănești region. A total of 88 samples (water, feed, raw milk, and dairy products) were seasonally collected during 2024–2025 and analysed for trace elements, geochemical fingerprint elements, and macroelements using ICP-OES and atomic absorption spectrometry. The strongest regional elemental contrasts were observed in groundwater for V (Δlog_10_ = +1.30), U (+0.82), Sr (+0.61), and Mn (+0.60), but these differences were substantially attenuated during biological transfer. Nevertheless, Li, U, Sr, Mn, and Cu retained measurable regional differences in milk. Regional elemental contrasts within the feed indicated an important inverse relationship (Pearson r = −0.60, *p* = 0.018), whereas no significant correlation was found between water and milk (Pearson r = −0.19, *p* = 0.493), suggesting that the milk elemental patterns may be influenced by biological regulatory processes in addition to the environmental signatures. Dairy processing further modified the elemental distribution, with the trace elements showing increased redistribution compared to the macroelements, while the geochemical fingerprint elements preserved regional information. The dietary exposure estimates for Cd and Ni remained below the corresponding toxicological reference values, whereas the highest estimated Pb exposure from hard cheese reached 0.977 μg/kg body weight/day for children and 0.558 μg/kg body weight/day for adults, exceeding the EFSA BMDL_01_ reference point. The results indicate that multielement fingerprinting may provide promising information for investigating elemental traceability and supporting dairy product authenticity assessment within the investigated production systems, although extensive geographical validation will be required before application as a general authentication tool.

## 1. Introduction

Milk and dairy products are key components of the human diet due to their nutrient composition. Their consumption varies worldwide, influenced by geographic location, culinary traditions, and economic and market factors [1]. Milk contains over 20 macro- and microelements. Some are essential in small amounts, including Fe, Zn, Cu, Mn, and Se, while others, such as Cr and Ni, are not required for human health [2,3].

Milk may also contain toxic elements, including Al, As, Cd, Pb, and Hg. These can cause kidney and liver damage, muscle atrophy, bone disorders, osteoporosis, hormonal and gastrointestinal problems, neurological and mental disorders, enzyme inhibition, impaired iron absorption, dysfunctions in the nervous, immune, reproductive, cardiovascular, and developmental systems, and metabolic disorders like anaemia [4,5].

Levels of highly toxic metals such as Cd, Hg, and Pb are generally low in raw milk, but environmental accumulation leads to their transfer into the food chain, increasing the risk of toxic effects in humans and animals [6,7]. Monitoring toxic elements in milk is necessary for consumer safety and risk assessment, although, in the European Union, maximum limits have only been set for Pb. Some studies in Croatia, Poland, and Serbia have reported Pb concentrations in milk exceeding the EU limit of 20 μg/kg [7,8,9]. EU Member States are required to monitor these metals under the National Residue Monitoring Plan, and EFSA compiles annual reports from all Member States [10]. Other countries have set limits for additional elements, such as As, Cr, and Pb in China [11,12].

Even essential elements like Cu, Fe, Se, and Zn can be toxic when present in excess. Excessive copper intake can cause Wilson’s disease due to ceruloplasmin deficiency [9]. Chronic Zn intake above 600 mg/day may cause neurological disorders linked to Cu deficiency [13]. High Fe intake (toxic threshold 200 mg) can damage the liver, heart, and other organs, while reducing the absorption of Cu and Zn [14]. Se is an essential trace element involved in antioxidant defence, thyroid hormone metabolism, and immune function through its incorporation into selenoproteins. Nevertheless, its nutritional benefits are associated with a relatively narrow range of adequate intake, and excessive exposure may result in adverse health effects, including selenosis [2]. Se at high concentrations can cause skin, nail, and bone disorders and is classified by IARC as Group 3, not classifiable as to its carcinogenicity [2]. Maintaining Se intake within the recommended range is important to guarantee the physiological benefits while avoiding toxicity [2]. Consequently, monitoring Se concentrations in foods of animal origin remains relevant for both nutritional evaluation and food safety.

While metals occur naturally in soil and the environment, their accumulation is largely driven by human activities such as industry, mining, wastewater discharge, traffic, agriculture, and livestock farming, as well as climatic factors [15,16]. These metals enter foods of animal origin, and consumer exposure depends on the type of food consumed. Element content in raw milk is influenced by the type of feed and water consumed by dairy animals, as well as by species, season, environment, and processing methods [17,18,19]. Studies show significant differences in levels of Al, Cd, Cu, Fe, Ni, Pb, and Zn between industrially contaminated and clean areas, and between urban and rural regions [20,21,22,23].

This study aimed to investigate the occurrence, transfer, and traceability of the essential elements (Ca, Mg, Na, K, Zn, Li, Cu, and Mn) and potentially toxic elements (Pb, Co, Ni, Cd, V, U, and Sr) along the water–feed–animal–milk chain in two contrasting Romanian dairy production systems, represented by a traditional region and an industrial region. The elemental concentrations were determined in the environmental inputs, raw milk, and dairy products to assess the propagation of the elemental signatures throughout the production chain and to explore their potential use as markers for geographical origin. Moreover, dietary exposure and health risks associated with milk consumption were evaluated through comparison with the international nutritional and toxicological reference values.

The novelty of the present study lies within the integrated assessment of the biological, environmental, seasonal and technological factors influencing elemental fingerprints within dairy production systems. While previous studies have mainly focused on the elemental contamination, food safety, or geographical differentiation of individual dairy products, the present investigation simultaneously evaluates feed, drinking water, milk, and cheese to assess the elemental transfer pathways throughout the feed–animal–milk chain. Moreover, this study integrates multielement fingerprinting with dairy processing transformation, seasonal variability, dietary exposure and health risk assessment, providing a comprehensive evaluation that correlates environmental exposure, product differentiation, and consumer safety. Finally, the comparative assessment of the traditional and industrial dairy production systems offers new insights into the preservation, attenuation, and redistribution of the elemental signatures and their potential use as complementary indicators supporting the geographical differentiation of dairy products.

## 2. Materials and Methods

### 2.1. Study Areas and Sampling Design

The study was conducted in two representative dairy-producing regions of Romania: Vaideeni (Vâlcea County) and Ibănești (Mureș County). The two regions were selected due to their contrasting environmental characteristics and dairy production systems. Vaideeni is characterized by traditional, small-scale livestock farming practices mainly based on local forage resources and artisanal dairy processing, whereas Ibănești represents a more industrialized dairy production system with increased technological standardization and centralized processing. These differences ensured a suitable framework to investigate the influence of environmental conditions and production practices on elemental transfer and traceability along the dairy chain.

Sampling was seasonally carried out during 2024–2025 to capture the potential temporal variations within the elemental composition. The sampling procedure was designed to evaluate the propagation of the elemental signatures from the environmental inputs to the milk and dairy products through the water–feed–animal–milk chain.

A total of 88 samples were collected from the representative dairy farms and processing units located in the two study regions. The samples included drinking water, feed components (hay, straw, cornmeal, and wheat bran), raw cow milk, and representative dairy products. The water samples were collected from the sources used for animal consumption, while the feed samples reflected the main dietary components provided to the dairy cattle during the study period. The water samples were collected in pre-cleaned polyethylene containers previously rinsed with deionized water and the corresponding sample. Immediately after collection, the samples were acidified with ultrapure HNO_3_ to pH below 2 to preserve the dissolved elements and subsequently stored at 4 °C until analysis. The experimental design was sample-based rather than farm- or animal-based, with each collected sample representing an analytical unit. The aim of the sampling strategy was to characterize the elemental composition of the different compartments of the water–feed–animal–milk production chain and to evaluate the propagation of regional elemental fingerprints across these matrices. Consequently, the study was designed to compare elemental signatures among matrices and regions rather than to assess the representativeness of individual farms or animal populations. Therefore, metadata such as the number of animals per farm, repeated sampling of individual cows, or the number of milk samples collected from each farm were not included in the sampling protocol established for this analytical investigation.

Sampling was performed using standardized procedures to guarantee the representativeness and comparability of the samples between the regions and seasons. The selected matrices were chosen because they represent the principal pathways through which essential and potentially toxic elements may enter the dairy production chain and subsequently influence the elemental composition of the milk and dairy products.

Raw milk samples were aseptically collected into sterile polyethylene containers, transported under refrigerated conditions (4 °C), and stored at −20 °C until sample preparation and instrumental analysis.

### 2.2. Sample Preparation

The reagents, including HCl (37%) and HNO_3_ (65% for analysis EMSURE), were purchased from Merck KGaA (Darmstadt, Germany). The ultrapure water was produced using an Elga Veolia PURELAB Flex3 system (Elga LabWater, High Wycombe, UK). All chemicals and reagents were of spectroscopic grade; the ICP Multi-Element Standard Solution IV Certipur, with a certified value of 1000.0 ± 20.0 mg/L (Merck KGaA (Darmstadt, Germany)), was used in the quantitative analysis for both trace and mineral elements for the calibration curve.

Water samples were acidified with 2% HNO_3_ prior to analysis.

Individual feed subsamples were pooled from the feed to obtain representative composite samples, air-dried at room temperature, and homogenized using a titanium knife mill (Grindomix GM 200, Retsch GmbH, Haan, Germany) to obtain a fine powder suitable for the elemental analysis. The feed, milk, and cheese samples were subjected to microwave-assisted acid digestion. For the milk, 5 mL was transferred into an 80 mL digestion vessel and mixed with 10 mL HNO_3_ (65%) and 3 mL HCl (37%). Aqua regia (HNO_3_/HCl) digestion was selected because it provides efficient dissolution of the mineral fraction and quantitative extraction of a broad range of trace and major elements from complex food and environmental matrices, ensuring accurate multielement determination by ICP-OES. The mixture was allowed to predigest for 15 min before the vessel was sealed. The microwave digestion program consisted of a 15 min ramp to 200 °C, followed by a 15 min hold at 200 °C. After controlled cooling, the vessels were opened, and the digests were filtered and diluted to 50 mL with deionized water [24]. All details regarding sample preparation are provided within Supplementary Material S1 (Tables S1–S5).

### 2.3. Analytical Procedure

Identification and quantification of the elements were performed using inductively coupled plasma optical emission spectrometry (ICP-OES; PlasmaQuant 9100, Analytik Jena GmbH, Jena, Germany). To minimize spectral and matrix interferences and improve analytical performance, the standard sample introduction system was replaced with inert components, including a ceramic D-torch and ceramic injector, combined with a PFA cyclonic spray chamber and a concentric nebulizer (OpalMist^®^ and DuraMist^®^, Glass Expansion, Melbourne, Australia).

The emission wavelengths were selected based on the maximum sensitivity and minimal interference, according to the established literature [25,26]. Quantification was carried out using external calibration with certified multielement standard solutions (Merck KGaA (Darmstadt, Germany). Calibration ranges were set at 5–100 µg/L for all samples. The concentrations obtained for the digested solutions were subsequently corrected for the digestion volume, sample mass, and any dilution factors, and expressed as mg/L for water samples and mg/kg (wet weight) for feed, milk, and cheese samples, as appropriate. Calibration curves exhibited excellent linearity, with correlation coefficients (R^2^) ranging from 0.99942 to 0.99998. Linearity was further verified using the Mandel test [27].

The instrumental parameters were optimized to guarantee analytical accuracy and reproducibility. The plasma gas (Argon) flow rate was maintained at 12.0 L/min, while the auxiliary and nebulizer gas flow rates were set at 0.7 L/min. The radio frequency (RF) power was fixed at 1200 W. The peristaltic pump flow rate was set to 1.1 mL/min, with a rinse solution flow rate of 1.5 mL/min and a rinsing time of 45 s to minimize carry-over effects. A read delay of 30 s and an integration time of 3 s per replicate were applied. All measurements were performed in triplicate. The element concentrations in feed samples were reported on a dry weight basis, while those in milk, cheese, and water were expressed on a fresh weight basis. The ICP-OES instrument parameters for multielement determination are provided within Supplementary Material S1 (Table S6).

Quality assurance and quality control (QA/QC) procedures included the analysis of reagent blanks, triplicate measurements, certified multielement calibration standards (ICP Multi-Element Standard Solution IV Certipur, Merck KGaA, Darmstadt, Germany), and spike recovery tests. The analytical methods demonstrated satisfactory performance, with recovery values ranging from 84.766% to 105.900% and good precision, as indicated by relative standard deviation (RSD) values below 10%. The methods were further validated by determining the limits of detection (LOD) and limits of quantification (LOQ) for all analysed elements, while procedures for correcting spectral interferences were also established. Detailed analytical validation parameters, including element-specific LOD, LOQ, calibration characteristics, and spectral interference corrections, are provided in Supplementary Material S1 (Table S7).

Identification and quantification of Ca, Mg, Na, and K were performed using flame atomic absorption spectroscopy (FAAS) with an Atomic Absorption Spectrophotometer NOVAA 300 (Analytik Jena GmbH, Jena, Germany) equipped with an air–acetylene flame. The fuel gas flow rate ranged from 0.8 to 4.0 L/min, while the support gas flow rate ranged from 13.5 to 17.5 L/min. Hollow cathode lamps specific to each element were used as radiation sources, and measurements were based on time-averaged absorbance. The resonance wavelengths employed were 422.7 nm (Ca), 285.2 nm (Mg), 589.0 nm (Na), and 766.5 nm (K). Lamp currents of 2–4 mA and a spectral bandwidth of 0.8–1.0 nm were selected according to the manufacturer’s recommendations (Supplementary Material S1, Table S8). Prior to each analytical series, calibration curves were established for each element within the appropriate mg/L concentration ranges. The AAS method was also validated by determining the element-specific LOD and LOQ values and by evaluating spectral interference correction procedures. Detailed validation data are provided in Supplementary Material S1 (Table S9).

### 2.4. Statistical Analysis and Data Visualization

Prior to statistical analysis, the analytical results were organized according to the production region (Vaideeni and Ibănești), sample matrix (water, feed, milk, and dairy products), and sampling season. For the feed, the concentrations determined in the individual feed components (hay, straw, cornmeal, and wheat bran) were combined to obtain a representative regional feed profile. For the dairy products that were common for both regions, the samples were grouped according to product category, including fresh cheese, brined hard cheese, and hard cheese.

The regional mean concentrations were subsequently calculated for each element and matrix. They were used to calculate the corresponding regional Δlog_10_ contrasts, which served as the basis for the elemental fingerprint analysis, propagation–attenuation assessment, transformation index calculations, Pearson and Spearman correlation analysis; seasonal variability evaluation, and dietary exposure assessment. This hierarchical grouping strategy allowed for the comparison of the elemental signatures across the environmental inputs, biological matrices, and processed dairy products, while minimizing the influence of individual sample variability.

Statistical analysis and graphical representations were performed using the Python programming environment (Python 3.12.3; Python Software Foundation, Wilmington, DE, USA). Data processing, management, and aggregation were conducted using the Pandas library (version 2.3.3), while the statistical computations were carried out using SciPy (version 1.13.1; NumFOCUS, Austin, TX, USA). Data visualization was performed using Plotly (version 6.3.1; Plotly Technologies Inc., Montreal, QC, Canada), enabling the generation of high-resolution graphical outputs. These tools were used to ensure a robust, reproducible, and transparent analysis of the elemental concentration variability throughout the dairy production chain. To investigate the potential relationships between the environmental inputs and milk composition, the correlation analysis was performed using the regional contrasts (Δlog10) calculated for each element.

#### 2.4.1. Elemental Fingerprint Propagation and Transformation Analysis

To investigate the propagation of elemental signatures along the environmental–animal–food continuum, regional elemental fingerprints were established for water, feed, milk, and dairy products. Rather than evaluating direct concentration ratios between matrices, the analysis focused on regional elemental contrasts and their evolution throughout the production chain, allowing for the assessment of the extent to which environmental signatures are preserved, attenuated, or modified during biological transfer and dairy processing.

For each analysed element, arithmetic mean concentrations were calculated separately for the two study regions (Vaideeni and Ibănești). Regional contrasts were expressed as logarithmic concentration differences according to the following equation:

(1) $${∆}_{{log}_{10}}={log}_{10}({\bar{C}}_{Ibănești})-{log}_{10}({\bar{C}}_{Vaideeni})$$

where ${\bar{C}}_{Ibănești}$ and ${\bar{C}}_{Vaideeni}$ represent the mean concentrations of a given element in samples collected from the Ibănești and Vaideeni regions, respectively.

Positive Δ_log10_ values indicate higher concentrations in samples originating from Ibănești, whereas negative values indicate enrichment in Vaideeni. The use of logarithmic differences allows for direct comparison among elements exhibiting concentration ranges spanning several orders of magnitude and facilitates visualization of regional elemental fingerprints.

To evaluate potential transfer pathways, the Δl_og10_ values obtained for milk were compared qualitatively and quantitatively with the corresponding Δl_og10_ values calculated for water and feed. Pattern similarity was assessed by examining both the direction (sign) and magnitude of the regional contrasts across elements.

To evaluate the transformation of elemental fingerprints during dairy processing, a geometric mean normalization approach was applied to allow for direct comparison between milk (expressed in mg/L) and dairy products (expressed in mg/kg).

For each product and region, a transformation index (T) was calculated according to Equation (2):

(2) $$T={log}_{10}\frac{\frac{C_{product}}{{GM}_{product}}}{\frac{C_{milk}}{{GM}_{milk}}}$$

where C_product_ and C_milk_ are the concentrations of a given element in the dairy product and corresponding milk sample, respectively, while GM_product_ and GM_milk_ represent the geometric mean concentrations calculated from the analysed elements in the dairy product and milk matrices.

This normalization minimizes matrix-dependent scaling effects and enables comparison of relative elemental distributions. Positive T values indicate relative enrichment of an element during processing, whereas negative T values indicate relative depletion.

To assess differences between production systems, the regional difference in transformation was calculated according to Equation (3):

(3) $$∆T=T_{Ibănești}-T_{Vaideeni}$$

Positive ΔT values indicate stronger relative enrichment in the industrial production system (Ibănești), whereas negative values indicate stronger relative enrichment in the traditional production system (Vaideeni).

To ensure a meaningful comparison of processing-induced elemental transformations between regions, the ΔT analysis was confined to the dairy products produced in both Vaideeni and Ibănești. The products specific to a single production system were not considered, as their inclusion would not allow for the direct attribution of observed differences to regional effects.

For interpretation purposes, the investigated elements were grouped into three categories according to their geochemical and biological behaviour: trace elements (Zn, Pb, Co, Cu, Ni, Cd, and Li), geochemical fingerprint elements (V, U, Sr, and Mn), and macroelements (Ca, Mg, Na, and K). This classification facilitates differentiation between environmentally driven signals and biologically regulated components of dairy composition.

#### 2.4.2. Pearson and Spearman Correlation Coefficients

Both Pearson and Spearman correlation coefficients were determined. Pearson correlation was used to evaluate linear associations between variables and was calculated according to Equation (4):

(4) $$r=\frac{\sum_{i}^{n}\left(x_{i}-\bar{x})(y_{i}-\bar{y}\right)}{\sqrt{\sum_{i}^{n}{\left(x_{i}-\bar{x}\right)}^{2}}\sqrt{\sum_{i}^{n}{\left(y_{i}-\bar{y}\right)}^{2}}}$$

where $x_{i}$, $y_{i}$ represent the paired observations of the two variables, $\bar{x}\;and$ $\bar{y}$ represent the mean values of the corresponding variables and r is the Pearson correlation coefficient.

Spearman rank correlation was additionally calculated to assess the monotonic relationship independently of the data distribution assumptions. Statistical significance was evaluated at *p* < 0.05. The correlation analysis was intended to evaluate the similarity of the regional elemental fingerprints between the environmental matrices and milk, and it was interpreted as a complementary indicator of the pattern similarity rather than as direct evidence of the biological transfer.

To investigate temporal variability in milk elemental composition, seasonal mean concentrations were calculated for each element and region. Because the analysed elements exhibited markedly different concentration ranges, data were standardized using z-score normalization according to Equation (5):

(5) $$z=\frac{x-\mu }{\sigma }$$

where x represents the seasonal mean concentration of an element, μ is the annual mean concentration, and σ is the corresponding standard deviation.

The z-score transformation expresses seasonal deviations relative to the annual average and allows for direct comparison among elements with different concentration ranges. Standardized values were visualized using heatmaps to identify seasonal enrichment and depletion patterns.

To evaluate the propagation of elemental signatures along the water–feed–milk continuum, comparative fingerprint diagrams based on Δlog10 values were generated. These visualizations allowed for simultaneous assessment of the magnitude and direction of regional contrasts across matrices.

The redistribution of elements during dairy processing was evaluated using the transformation index (T) and the regional transformation difference (ΔT), as described in Section 2.4. Comparative heatmaps and grouped graphical representations were used to visualize elemental enrichment and depletion patterns among dairy products and production systems.

All graphical outputs were generated using standardized plotting procedures to facilitate comparison among matrices, products, seasons, and elemental groups.

### 2.5. Dietary Exposure and Risk Assessment

A screening-level dietary exposure assessment was performed by calculating the estimated daily intake (EDI) of Pb, Cd, and Ni through the consumption of milk and dairy products. The estimated daily intake values were subsequently compared with the corresponding health-based guidance values to evaluate whether the measured concentrations approached levels of toxicological concern. For the essential elements, including Cu, Zn, and Mn, the EDI was calculated as follows [28,29,30]:

(6) EDI=C×MS

where C is the concentration of the element in milk (mg/kg, fresh weight) and MS is daily milk consumption (g/day). For potentially toxic elements such as Pb, Ni, and Cd, EDI was normalized to body weight using the following equation [28,29,30]:

(7) EDI=(C×MS)/BW

where BW represents body weight (kg).

Two consumption scenarios were considered: average consumption and high consumption (95th percentile, P95). The average milk consumption rate was assumed to be 216.2 g/day, while the high-consumption scenario was represented by 511.8 g/day, according to the available dietary intake data. Body weight-normalized consumption rates were 2.96 and 7.27 g/kg body weight day, respectively.

For the elements associated with cumulative exposure, the estimated weekly intake (EWI) was calculated according to the following [28,29,30]:

(8) EWI=EDI×7

The calculated exposure values were compared with the toxicological reference values established by international regulatory authorities, including the Tolerable Daily Intake (TDI), Tolerable Weekly Intake (TWI), Benchmark Dose Lower Confidence Limit (BMDL), Provisional Maximum Tolerable Daily Intake (PMTDI), and Tolerable Upper Intake Level (UL), where applicable [10,31,32,33,34].

## 3. Results and Discussion

The complete dataset comprising the sampling and seasonal distribution, as well as the elemental concentrations determined in water, feed, milk, and dairy product samples collected from the Vaideeni and Ibănești regions, is provided in Supplementary Material S2. These data served as the basis for the subsequent statistical analysis, fingerprinting evaluations, transformation index calculations, seasonal variability assessments, and dietary exposure estimations.

### 3.1. Regional Elemental Fingerprints of Water and Feed

**Figure 1:**
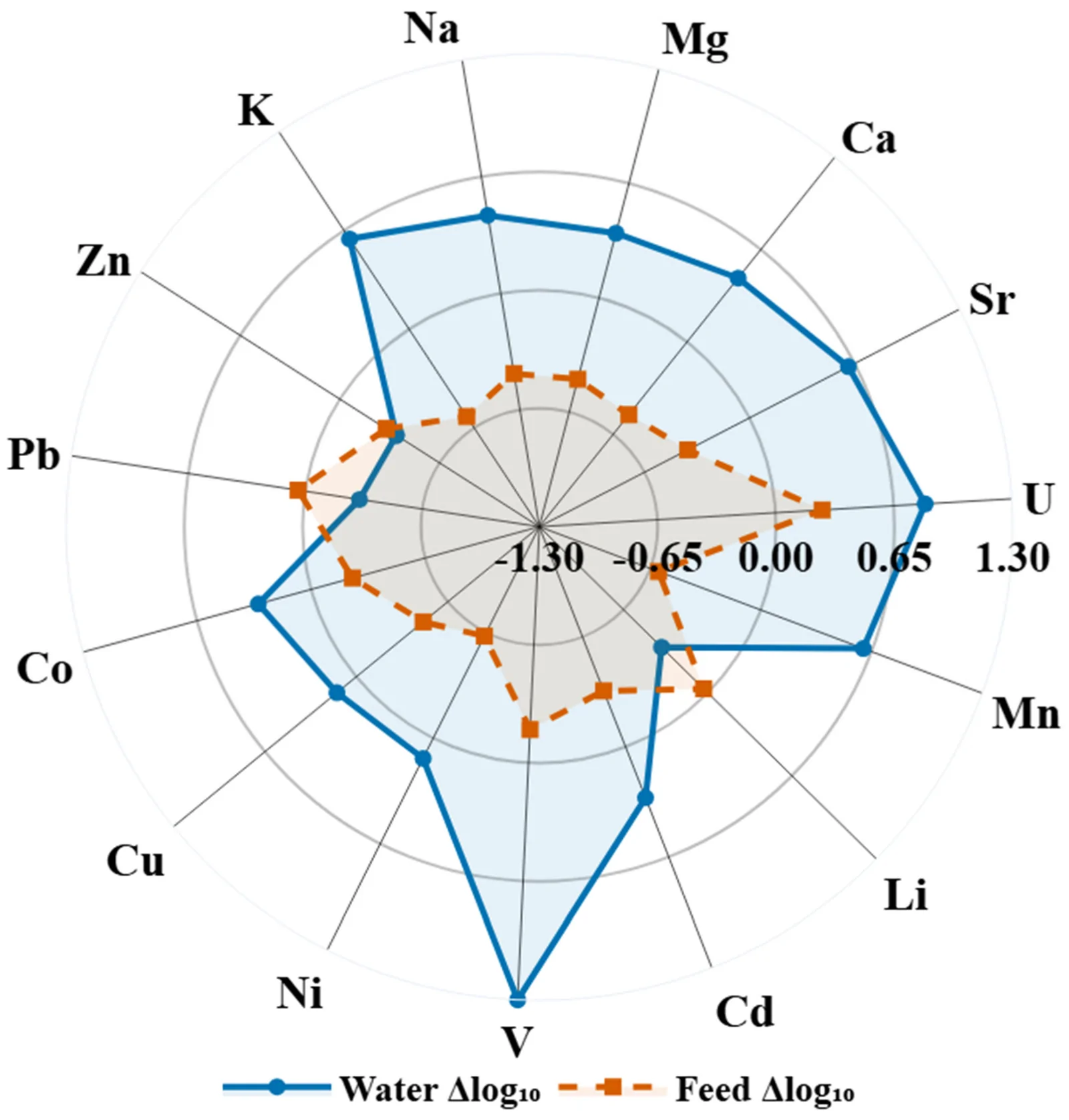
Input fingerprint—Δlog_10_ (Ibănești vs. Vaideeni).

The elemental composition of water and feed exhibited distinct regional concentration patterns between the Vaideeni and Ibănești dairy production systems. These regional concentration patterns were consistent with variation in the environmental conditions influencing the initial stages of the dairy production chain. The regional mean concentrations and corresponding Δlog10 values are summarized in Table 1, while the overall elemental fingerprints are visualized in Figure 1. The radar plots were intended as descriptive visualization tools that facilitate comparison of regional mean Δlog10 patterns across matrices and should not be interpreted as statistical evidence of regional differentiation.

Taken together, these descriptive concentration patterns may reflect, at least in part, differences in hydrogeochemical and agricultural conditions between the two investigated dairy production systems.

It may be observed from Table 1 that the groundwater samples from Ibănești were systematically enriched in several geochemically relevant elements, including V (Δlog10 = +1.30), U (+0.82), Sr (+0.61), Mn (+0.60), and K (+0.59), while moderate enrichments were observed for Ca, Mg, and Na. In contrast, the water samples from Vaideeni contained higher concentrations of Zn (Δlog10 = −0.37) and Li (−0.36). The magnitude of these regional concentration differences is consistent with the variation in groundwater composition between the two regions and may reflect differences in geological substrate, mineral weathering processes, groundwater residence times, and local hydrogeological conditions [35,36].

The simultaneous enrichment of V, U, Sr, and Mn in Ibănești is of particular interest, because these elements are commonly associated with the lithological controls and hydrogeochemical processes [37]. Similar associations have been reported in groundwater systems where trace element mobility is governed by the bedrock composition, mineral dissolution, and redox conditions [38,39]. The coherent behaviour observed for these elements was, therefore, consistent with the geogenic influence on the regional water composition, although the present study did not directly investigate the geological or hydrogeochemical characteristics of the study areas.

The hydrogeochemical fingerprint is more clearly visualized in Figure 1, where the water profile displays a pronounced outward expansion for most geochemical and macroelements. The coherent shape of the water fingerprint illustrates a common regional concentration pattern across several elements. This descriptive pattern is consistent with a region-specific groundwater signature, although no direct hydrogeological investigation was performed in the present study. Similar multielement patterns have been proposed as effective indicators of geographical origin in food authentication studies [40,41].

The feed samples exhibited a different elemental fingerprint. As Table 1 reveals, the strongest regional contrasts were observed for Ni (Δlog10 = −0.63), Mn (−0.60), K (−0.58), Ca (−0.51), Mg (−0.46), and Cu (−0.48), all indicating higher concentrations in Vaideeni feed. Only some elements, mainly U (+0.26), had enrichment in Ibănești. Overall, the feed matrix exhibited lower-amplitude regional contrasts than the water and a substantially different direction for several elements. The feed fingerprint presented in Figure 1 further illustrates this divergence. Unlike the relatively coherent hydrogeochemical profile that was observed for the water, the feed profile was more irregular and attenuated, suggesting the combined influence of the environmental, biological, and agricultural factors. In addition to the soil geochemistry, the elemental composition of forage may be governed by element-specific plant uptake, botanical composition, soil physicochemical properties, fertilization practices, harvesting conditions, or feed formulation. These factors may selectively enrich or deplete the individual elements, partially masking the underlying geochemical signature of the production area. Similar findings have been reported in studies investigating the elemental variability in forage crops and dairy feed mixtures, where agricultural management practices exerted a stronger influence on the elemental composition than the local hydro-geochemistry [8,9].

The contrasting fingerprints observed in Figure 1 indicate the coexistence of two distinct environmental control mechanisms within the studied dairy systems. The water composition appears to be mainly governed by the hydrogeochemical factors, whereas the feed composition reflects agricultural management and biomass sourcing. The limited overlap between the two fingerprints suggests that the elemental transfer within the dairy chain may be governed by multiple, partially interconnected pathways, with the groundwater mainly reflecting the regional geochemistry and feed integrating both natural and management-related influences before the biological transfer to animal-derived products.

From a traceability point of view, these findings are relevant because they indicate that the regional differentiation was already established at the environmental input level. Previous studies postulated that the water and feed may represent the main sources for the elemental variability in dairy production systems and may serve as the basis for geographical discrimination of milk and dairy products [40,42]. Therefore, the combined evaluation of these environmental matrices may provide the basis for investigating how the elemental signature may be selectively transferred, attenuated, or modified during plant uptake, animal metabolism, and subsequent dairy processing. Understanding these processes is essential for assessing the extent to which environmental fingerprints are preserved throughout the feed–animal–milk chain and their potential application as complementary indicators of geographical differentiation.

### 3.2. Milk Elemental Composition and Environmental Traceability

**Figure 2:**
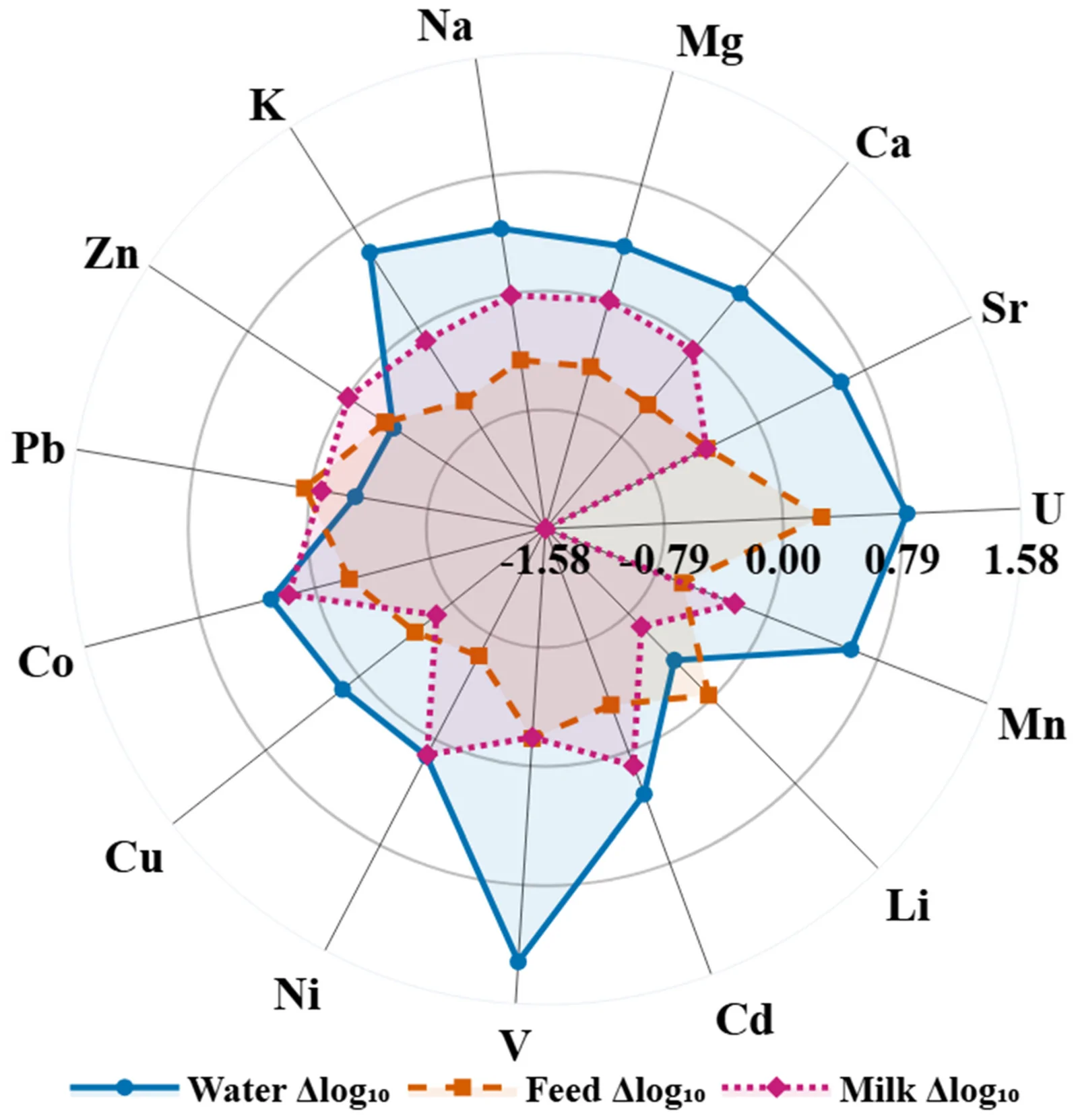
Comparative regional Δlog_10_ (water, feed, milk)—Ibănești vs. Vaideeni.

The milk elemental composition had a lower regional variability than that observed in either water or feed. This descriptive pattern is consistent with the influence of physiological regulatory mechanisms controlling the absorption, distribution, and secretion of mineral elements into the dairy matrix. Although several elements displayed clear regional concentration differences in the environmental matrices, these contrasts were generally attenuated in milk, resulting in a comparatively smoother elemental fingerprint (Table 2; Figure 2).

Among the analysed elements, the most pronounced regional contrasts in the milk were observed for U (Δlog10 = −1.58), Li (−0.67), Cu (Δlog10 = −0.66), Sr (Δlog10 = −0.39), and Mn (Δlog10 = −0.23), indicating higher concentrations for Vaideeni. Only few elements revealed an enrichment in Ibănești milk, namely Co (Δlog10 = +0.18), Ni (Δlog10 = +0.12), and Cd (Δlog10 = +0.10). The macroelements Ca, Mg, Na, and K had Δlog10 values close to zero, despite the substantial differences within the corresponding water and feed matrices. This suggests that the mechanisms controlling elemental incorporation into the milk were independent from the environmental concentration gradients. This behaviour may reflect the tight homeostatic regulation of the essential macroelements, whose concentrations in milk were maintained within relatively narrow physiological ranges to secure adequate neonatal nutrition and normal mammary gland function [35,40].

The most striking example was represented by U. While the groundwater from Ibănești had a strong positive regional contrast (Δlog10 = +0.82), the milk exhibited a substantial negative contrast (Δlog10 = −1.58), corresponding to about two orders of magnitude difference between the environmental and biological matrices. A similar inversion was observed for Sr, which was enriched in Ibănești water (Δlog10 = +0.61) but displayed higher concentrations in Vaideeni milk (Δlog10 = −0.39). These findings indicate that the increased environmental availability did not necessarily translate into proportional transfer to milk, highlighting that environmental availability alone may be insufficient to predict the elemental concentrations within the milk because biological barriers regulate the transfer efficiency of the individual elements.

Previous studies showed that the transfer of trace elements from environmental sources to milk may be influenced by a combination of gastrointestinal absorption efficiency, ruminal metabolism, blood–mammary transport, and homeostatic regulation [35,41,42]. Ca, Mg, K, and Na may be subject to strict physiological control because of their essential roles in cellular metabolism, osmotic regulation, and milk secretion. As a result, even pronounced differences in environmental exposure may result in only minor variation in milk composition, mainly for the nutritional essential elements whose secretion is actively regulated by the mammary gland. Similar observations were reported in the literature, indicating relatively stable concentrations of the major elements within milk despite differences in the environmental conditions and feeding systems [40,43]. This interpretation is also consistent with studies conducted in neighbouring Central and Eastern European countries. Some investigations performed in Croatia and Poland similarly reported that, despite the regional differences in the environmental conditions and farming practices, the concentrations of the essential macroelements in milk remained relatively stable, whereas increased variability was generally observed for the selected trace elements, reflecting the combined influence of environmental exposure and physiological regulation [2,7,8].

The attenuation effect was not limited to the macroelements. Several trace elements exhibiting substantial variability in feed also showed only weak differentiation in milk. For example, the feed samples from Vaideeni contained considerably higher concentrations of Ni, Mn, and Cu compared to those from Ibănești, yet the corresponding differences in the milk were reduced. The observed attenuation suggests that the efficiency of elemental transfer differed among the trace elements, depending on their absorption, tissue retention, and mammary secretion. This behaviour is consistent with some previous reports indicating that the mammary gland may act as a selective barrier, restricting the transfer of many trace elements into the milk even when the environmental exposure differs substantially [41,43].

An interesting aspect was that certain elements retained a detectable regional signature despite the biological regulation. Li, U, and Sr exhibited the most pronounced regional differences among the geochemical fingerprint elements, suggesting that these elements may represent promising complementary indicators for regional differentiation when interpreted in combination with the multielement fingerprints. Similar conclusions have been reported in food authentication studies, where Sr isotopes and trace element patterns were successfully applied as geographical markers due to their partial resistance to biological homogenization [40,41].

The relatively small regional differences observed for Zn, Ca, Mg, and Na highlight that these elements are unlikely to provide robust regional differentiation on their own. Although these elements evidence an individual limited discriminatory power because of the physiological regulation, they may still improve multivariate classification models by contributing complementary information when evaluated together with the more region-sensitive trace elements. On the contrary, the stronger differentiation observed for Li, U, Sr, Cu, and Mn suggested that these elements may represent informative components of the regional elemental fingerprint and warrant further investigation as potential complementary indicators in future authentication studies.

The overall milk fingerprint had an intermediate position between the environmental signatures of the water and feed (Figure 2). It neither reproduced the hydrogeochemical fingerprint of the groundwater nor directly reflected the elemental composition of the feed. Instead, it can be viewed as a biologically filtered integration of the multiple environmental inputs. This biological filtering may reduce the direct environmental variability while preserving part of the regional elemental signature, thereby explaining the intermediate position of the milk between the environmental inputs and processed dairy products. This observation is consistent with the hypothesis that milk should be regarded as a regulatory interface within the dairy chain, where the environmental information may be selectively preserved, attenuated, or transformed before being transferred to processed dairy products.

From a traceability perspective, these findings show that, although the physiological regulation substantially attenuated the environmental variability, it did not completely eliminate the regional elemental differences. The persistence of the contrasts between the selected trace and geochemical elements indicates that part of the environmental signature was preserved throughout the biological transfer, providing a mechanistic basis for investigating whether these fingerprints remained detectable after dairy processing. This observation may support the use of the multielement fingerprint as a complementary tool for regional differentiation when interpreted together with the environmental and production-related information.

### 3.3. Propagation of Elemental Signatures Along the Water–Feed–Milk Chain

The comparison of the regional elemental contrasts across the water, feed, and milk matrices highlighted a clear propagation–attenuation pattern along the environmental–biological continuum. Although substantial regional differences were observed in the environmental inputs, these contrasts were progressively reduced within the dairy matrix, suggesting that the elemental transfer from the environment to milk was non-conservative and strongly influenced by the physiological regulation, including element-specific absorption, tissue distribution, metabolic use, and mammary gland secretion. As a result, the environmental differences became progressively attenuated before being expressed within the dairy matrix.

**Figure 3:**
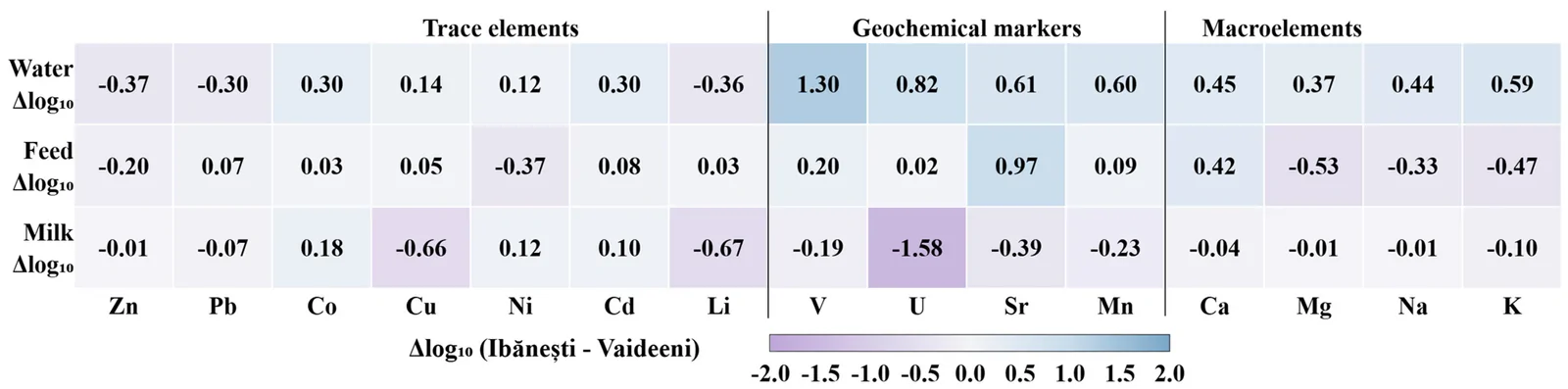
Transfer map of regional elemental contrasts.

The transfer map presented in Figure 3 provides an integrated overview of the regional elemental variability throughout the production chain.

Water exhibited the strongest and most coherent regional contrasts, mainly for V, U, Sr, Mn, and the major ions (Ca, Mg, Na, and K). These elements formed a distinct hydrogeochemical cluster characterized by consistent positive Δlog10 values in Ibănești, reflecting the higher mineralization of the groundwater within this region. In constrast, the feed displayed a different pattern, with negative contrasts dominating for Ni, Mn, K, Ca, Mg, and Na, indicating higher concentrations within the Vaideeni feed. The coexistence of these contrasting fingerprints evidences that the groundwater chemistry and agricultural management may represent independent drivers of elemental variability within the dairy production system [35,37]. Because the groundwater chemistry mainly reflects the geological processes whereas the feed composition integrates both environmental and management-related influences, the two matrices may provide complementary rather than redundant information on the origin of elemental variability.

Despite these evident environmental differences, the milk presented considerable attenuated contrasts for most elements. Several elements that exhibited strong enrichment in the water, including V, U, and Sr, showed only weak differentiation within the milk. The elements exhibiting substantial feed-related contrasts did not proportionally propagate into the dairy matrix. This attenuation indicates that the environmental variability was filtered by the biological processes operating at several levels, including gastrointestinal absorption, systemic distribution, metabolic regulation, and selective transport across the mammary epithelium. Consequently, only part of the environmental elemental signature was preserved in milk, resulting in a smoother elemental profile. Similar attenuation effects have been reported for the transfer of trace elements from soil, water, and feed into the milk, where biological regulation significantly reduced the magnitude of the environmental signals [35,37,40,41].

The correlation analysis was performed using the Δlog10 values calculated for all elements to quantitatively evaluate the similarity between the environmental and milk fingerprints (Table 3).

The Spearman correlation analysis indicated weak and statistically non-significant relationships between the water and milk (ρ = −0.35, *p* = 0.196) and between the feed and milk (ρ = −0.26, *p* = 0.348). These results suggest that the magnitude of the regional elemental differences observed within the environmental matrices cannot be directly extrapolated to milk composition and are consistent with the interpretation that element-specific biological regulation modified the environmental signals during the transfer through the dairy chain.

The Pearson correlation analysis resulted in similar weak results for the water versus milk (r = −0.19, *p* = 0.493), consistent with the absence of a direct linear relationship between the groundwater composition and milk elemental profiles. In contrast, the feed versus milk exhibited a moderate negative linear correlation (r = −0.60, *p* = 0.018), suggesting that the regional differences in feed composition may be associated with the observed regional variability within the milk chemistry. Nevertheless, the negative correlation suggested that this relationship was not governed by a direct proportional transfer but rather by element-specific physiological regulation.

The discrepancy between the Pearson and Spearman coefficients was informative. While the statistically significant Pearson correlation suggested an association between feed-related elemental differences and the observed regional variability in milk composition, the absence of a corresponding significant Spearman correlation indicated that this relationship was not uniformly expressed across all elements. Instead, the individual elements appeared to exhibit different transfer behaviours, consistent with element-specific physiological regulation. These observations suggest that each element may differently respond to biological regulation, making the multielement approaches more informative than the interpretation based on individual elements alone. Such element-specific responses have been previously reported for both essential and non-essential elements, reflecting differences within bioavailability, intestinal absorption, metabolic requirements, and excretion pathways [8,40].

Several examples evidenced this selective transfer behaviour. For example, Ni had one of the strongest regional contrasts in the feed (Δlog10 = −0.63) but only a modest contrast in milk (Δlog10 = +0.12). Mn exhibited a pronounced difference in the feed (Δlog10 = −0.60) but a substantial attenuated difference in the milk (Δlog10 = −0.23). In contrast, U exhibited limited regional contrast in the feed but one of the highest regional contrasts within the milk (Δlog10 = −1.58). These examples highlight that environmental availability alone cannot explain the observed milk composition and emphasize the importance of physiological regulation mechanisms. Together, these examples show that the efficiency of elemental transfer was highly element-specific and cannot be predicted only from environmental abundance. The chemical properties, physiological requirements, and biological regulation collectively determined the extent to which the individual elements were incorporated into the milk.

The combined interpretation of Figure 3 and Table 3 supports the hypothesis of an existing propagation–attenuation model for elemental transfer within the dairy chain. The environmental signatures were initially established in the water and feed, then modified by the biological processes during incorporation into the milk, and ultimately expressed as the attenuated elemental fingerprints. This behaviour is consistent with the concept of the milk as a biological integration matrix that may selectively preserve certain environmental signals while suppressing others [9,41]. This integrative role may explain why milk simultaneously preserves part of the regional environmental information while exhibiting substantially lower elemental variability compared to the original environmental matrices.

From a traceability point of view, the results indicate that the observed regional differences were unlikely to be explained only by the absolute elemental concentrations or simple environmental–milk relationship. Instead, the multielement fingerprints capturing the residual environmental signal after biological filtering may provide more robust complementary markers of the regional origin. Previous studies reported similar conclusions for food authenticity using the elemental and isotopic approaches, where a combination of markers consistently outperformed individual elements for provenance determination [42,43].

Overall, the propagation analysis evidenced that the environmental elemental signatures may be transferred through the dairy chain in a non-conservative manner. Although substantial attenuation occurred during the biological transfer, selected elements retained measurable regional contrasts, indicating that some aspects of the regional elemental pattern persisted across the production chain. These findings provided a conceptual framework for understanding how the environmental signatures evolve throughout the dairy production chain and establish a basis for evaluating the additional modification introduced during dairy processing.

### 3.4. Transformation of Elemental Fingerprints During Dairy Processing

While the previous sections showed that milk represents a biologically regulated integration of environmental inputs, dairy processing introduced an additional level of complexity by modifying the distribution of elements among the product fractions. Processes such as coagulation, whey separation, fermentation, salting, maturation, smoking, and fat concentration may alter the physicochemical environment of the milk constituents and, therefore, may influence the partitioning behaviour of the individual elements. Therefore, the processed dairy products cannot be considered simple concentration products of milk but rather transformed matrices in which elemental redistribution results from the combined effects of technological processing and element-specific physicochemical properties.

**Figure 4:**
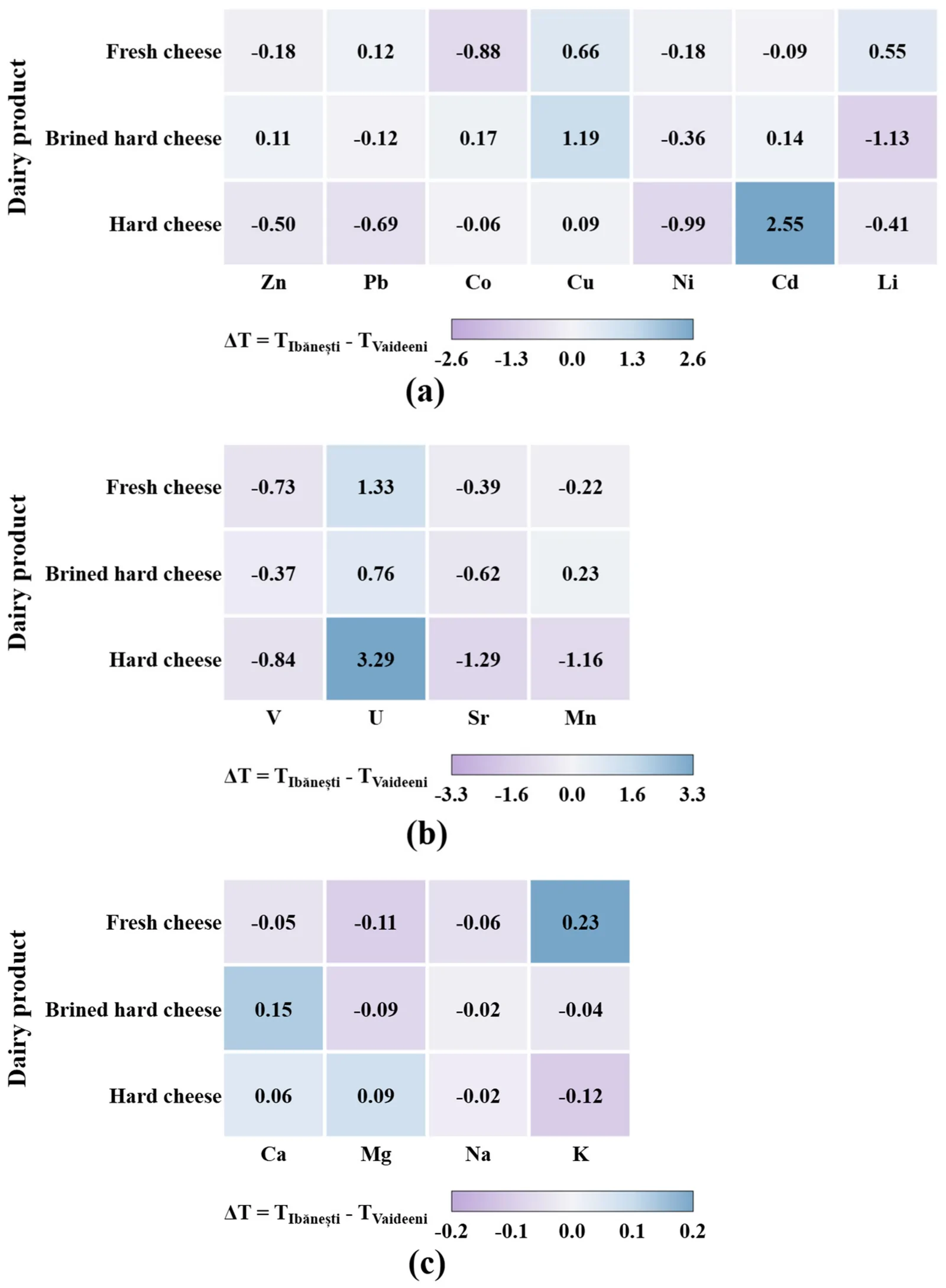
Regional differences in milk-to-product elemental transformation (∆T) between Ibănești and Vaideeni for (**a**) trace elements, (**b**) geochemical fingerprint elements, and (**c**) macroelements.

To evaluate these transformations, the regional differences in milk-to-product enrichment were assessed using the transformation index (T) and the corresponding regional transformation difference (ΔT). The positive ΔT values indicate stronger relative enrichment in the products originating from the industrial Ibănești system, whereas the negative values indicate stronger enrichment in the traditional Vaideeni system. The transformation behaviour of the three elemental groups is separately illustrated in Figure 4a (trace elements), Figure 4b (geochemical fingerprint elements), and Figure 4c (macroelements). The comparison presented in Figure 4a–c is restricted to the dairy products available for both regions. Products represented only in a single production system were excluded from the regional comparison to guarantee direct comparability between Vaideeni and Ibănești.

#### 3.4.1. Transformation of Trace Elements

The strongest processing-related effects were observed for the trace elements, as illustrated in Figure 4a. This group had the largest range of ΔT values and the most pronounced product-specific responses, indicating that trace elements may be sensitive to the technological processing conditions. Several elements exhibited substantial enrichment in specific cheese categories, reflecting differences in their affinity for the protein-rich curd fractions, aqueous whey phases, and fat-containing matrices.

Among the analysed elements, Cd exhibited one of the most pronounced positive ΔT values, mainly in the hard cheese, indicating stronger enrichment in the products originating from the industrial Ibănești system. Cu displayed a similar trend, with consistent positive ΔT values across several cheese categories, mainly in brined hard cheese. These patterns suggest that the industrial processing conditions, together with the element-specific physicochemical properties, may favour the retention of the specific trace elements during coagulation and maturation.

On the contrary, Zn and Ni generally had negative ΔT values (Figure 4a), indicating relatively stronger enrichment in the products manufactured within the traditional Vaideeni system. Such differences may reflect variation in milk composition or technological processing conditions (including moisture reduction, curd handling, salting, and maturation), together with the element-specific physicochemical properties governing trace element partitioning. The existing literature has reported similar redistribution phenomena during cheese manufacture, with trace metal retention being shown to strongly depend on whey drainage efficiency and casein-associated binding mechanisms [35,37].

Overall, trace elements were the most responsive group to dairy processing, therefore representing the component of the elemental fingerprint most susceptible to technological modification [44].

#### 3.4.2. Transformation of Geochemical Fingerprint Elements

The transformation behaviour of geochemical fingerprint elements is presented in Figure 4b. Contrary to the high variable patterns observed for trace elements, this group exhibited more coherent and systematic responses across the dairy products. Because these elements were mainly related with the environmental and geological signatures, their behaviour during processing was relevant for traceability applications.

U displayed consistently positive ΔT values, particularly within the hard cheese and other concentrated dairy products, suggesting preferential enrichment in the products originating from the industrial production system. V and Sr generally presented negative ΔT values, indicating stronger enrichment in the traditional products. These contrasting responses highlight that the environmental signatures were not simply preserved during processing but they were selectively modified according to the element-specific partitioning mechanisms.

However, the persistence of clear regional differences across several product categories evidenced that processing did not completely delete the environmental information. Elements such as U, Sr, and V continued to present distinguished regional patterns after the transformation of milk into cheese products. Similar observations have been reported in different studies, where the geochemical-derived elements retained geographical information despite extensive technological processing [18,41].

The results presented in Figure 4b suggest that the geochemical fingerprint elements may constitute the most robust group of markers for geographical origin determination, combining environmental sensitivity with relatively good stability during processing.

#### 3.4.3. Transformation of Macroelements

The transformation behaviour of the macroelements is presented in Figure 4c. Contrary to both trace and geochemical elements, Ca, Mg, Na, and K presented comparable small ΔT values across all dairy products, indicating limited regional differences in processing-related enrichment.

This stability reflects the strong physicochemical constraints governing the distribution of major milk constituents. Ca and Mg were extensively associated with casein micelles; therefore, they followed relative predictable partitioning pathways during coagulation and curd formation. Na and K were mainly dissolved in the aqueous phase and were influenced by moisture content and whey removal. This was the reason why these elements appeared less sensitive to the regional processing differences than either trace or geochemical elements [45].

The relatively narrow ΔT ranges indicate that the processing behaviour of the macroelements was similar in both production systems. This observation was consistent with the influence of common dairy processing mechanisms, although the present study did not directly investigate the involved technological factors. Although this may limit their use as individual geographical markers, their stability may contribute to the reproducibility of the multielement fingerprint.

#### 3.4.4. Implications for Traceability

The observed differences between the traditional and industrial production systems provided additional insight into the role of technological standardization. The products from Vaideeni were primarily manufactured through the artisanal processing practices at the farm level, whereas the products from Ibănești were produced under industrial conditions involving intense process control and standardization. The ΔT analysis presented in Figure 4 indicates that the processing scale itself may influence elemental redistribution. Industrial production appeared to promote stronger enrichment of the selected elements (Cd, Cu, or U), whereas traditional production favoured the retention of others, including Zn, Ni, V, and Sr.

These findings support the growing recognition that food traceability cannot exclusively rely on environmental inputs, but must also consider processing-related transformations [40,46]. The elemental composition of a dairy product represents the cumulative result of the environmental exposure, biological regulation, and technological modification. Therefore, dairy products should be considered dynamic matrices in which the elemental fingerprints transform throughout the production chain, rather than passive repositories of the original milk composition.

Overall, the ΔT analysis indicates that dairy processing acts as an active and selective filter able to amplify, attenuate, or redistribute the elemental signatures inherited from milk. The trace elements presented the highest sensitivity to processing, geochemical fingerprint elements retained an important part of their regional information despite redistribution, and macroelements remained predominantly stable. These results evidence the importance of considering technological transformations when developing elemental fingerprinting approaches for dairy product authentication and geographical origin determination.

### 3.5. Seasonal Variability of Elemental Fingerprints

The evaluation of seasonal variability provided important information regarding the temporal stability of the regional elemental fingerprints and their potential suitability for geographical traceability applications. An informative elemental marker should not only exhibit regional differences between production systems, but should also remain sufficiently stable throughout the year. To investigate this, seasonal trends were evaluated for the geochemical fingerprint elements and subsequently quantified using a seasonal variability index.

**Figure 5:**
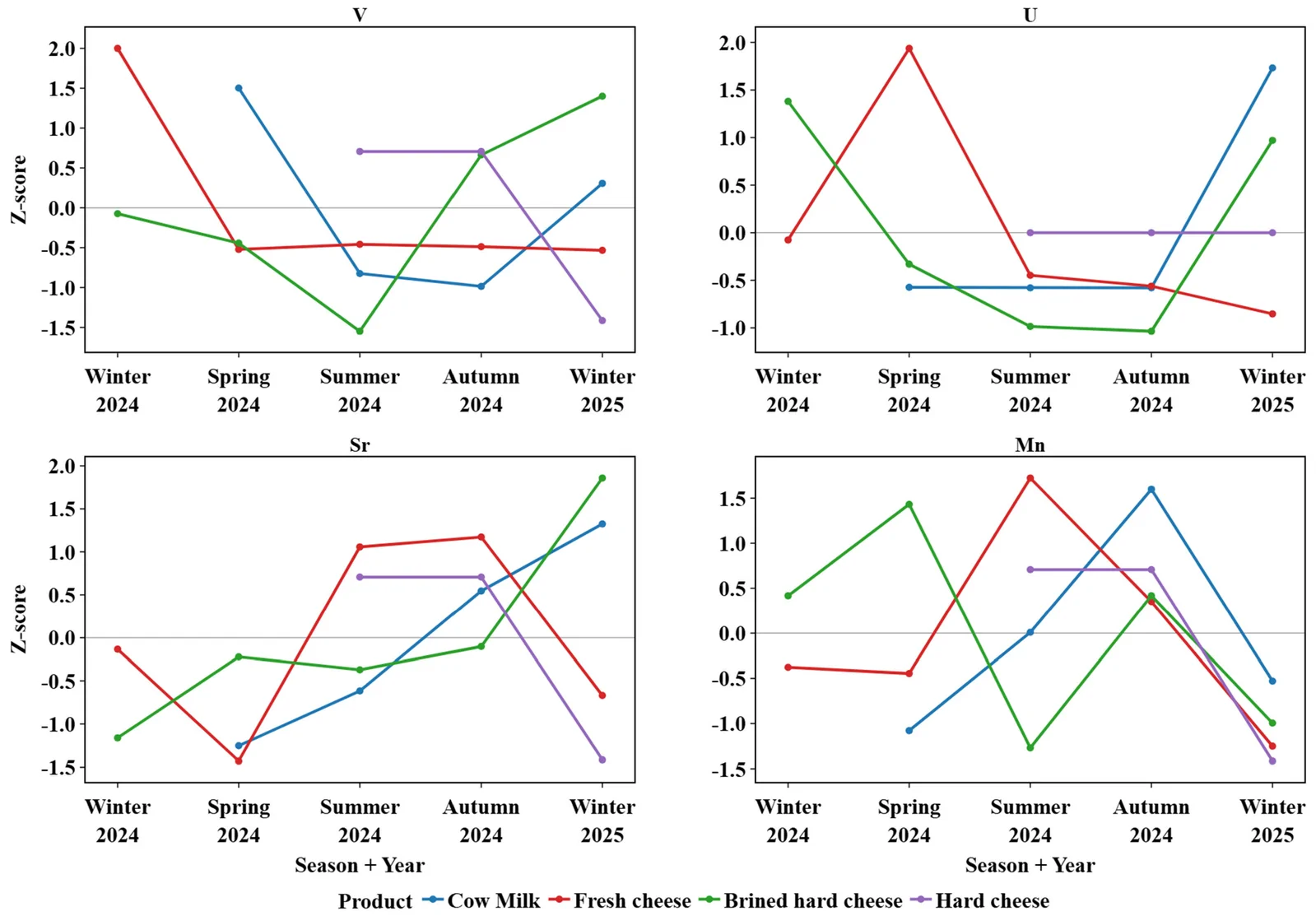
Seasonal evolution of geochemical fingerprint elements in Vaideeni.

The seasonal evolution of the geochemical fingerprint elements in the dairy products originating from Vaideeni is presented in Figure 5.

Overall, the results indicate relatively moderate seasonal fluctuations for V, U, Sr, and Mn, with no consistent temporal trend shared across all dairy products. In contrast, the observed variability is strongly product-dependent, suggesting that the seasonal influences from the environmental inputs were partially preserved during milk processing but were subsequently modified by technological factors.

Certain products exhibited transient enrichments for the specific elements during particular seasons, whereas others remained comparative stable throughout the year. For example, Sr and Mn exhibited seasonal fluctuations in several cheese products, while U displayed a more stable distribution. These observations suggest that the transfer of environmental information through the dairy chain was not only influenced by biological regulation, but also by product-specific partitioning processes that occurred during coagulation, whey separation, and maturation. Some dairy traceability studies reported similar findings, where seasonal influences were detected but generally weaker than geographical effects [40,41].

**Figure 6:**
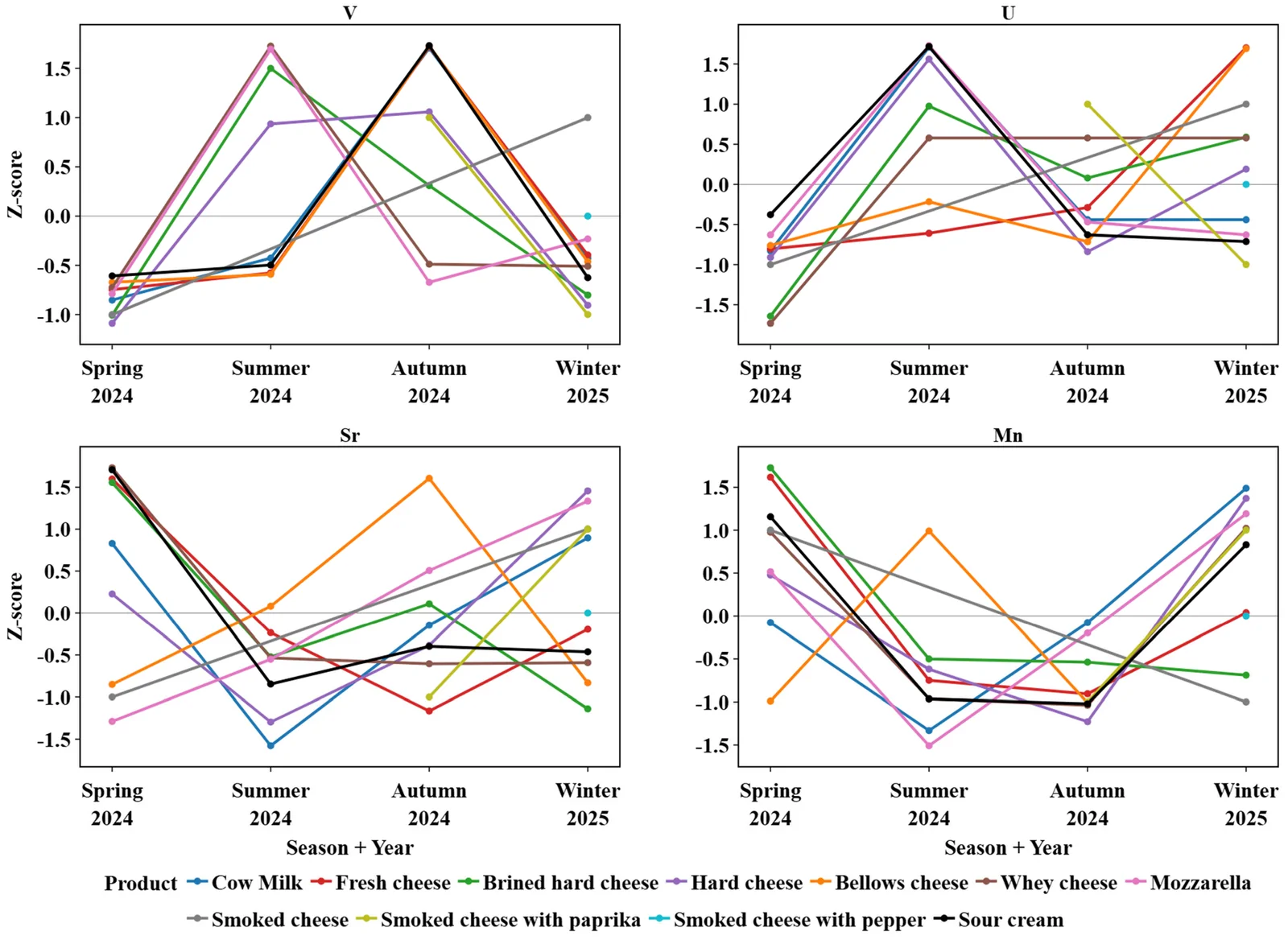
Seasonal evolution of geochemical fingerprint elements in Ibănești.

The seasonal behaviour of the geochemical fingerprint elements in the products from the Ibănești production system is presented in Figure 6.

Compared with Vaideeni, the Ibănești products presented increased temporal variability, mainly for V and U. More pronounced fluctuations were observed among the processed dairy products, suggesting that the combination of seasonal environmental variation and industrial-scale processing may contribute to the redistribution of elemental signatures.

The patterns evidenced in Figure 6 further suggest that industrial processing may influence the temporal expression of the elemental fingerprints. Unlike the artisanal products from Vaideeni, which preserved the local environmental characteristics, industrial production involved milk pooling, batch standardization, and process control measures that may modify or redistribute the elemental signatures. Similar effects were observed in other studies, where blending and technological standardization influenced the expression of geographical markers [46,47].

Additional seasonal trends for the trace elements and macroelements are provided in Supplementary Material S1 (Figures S1–S4). These results suggest that the seasonal effects were generally secondary compared with the product-specific and processing-related influences. Although temporal fluctuations were observed for certain trace elements, no universal seasonal pattern emerged across all dairy products.

**Figure 7:**
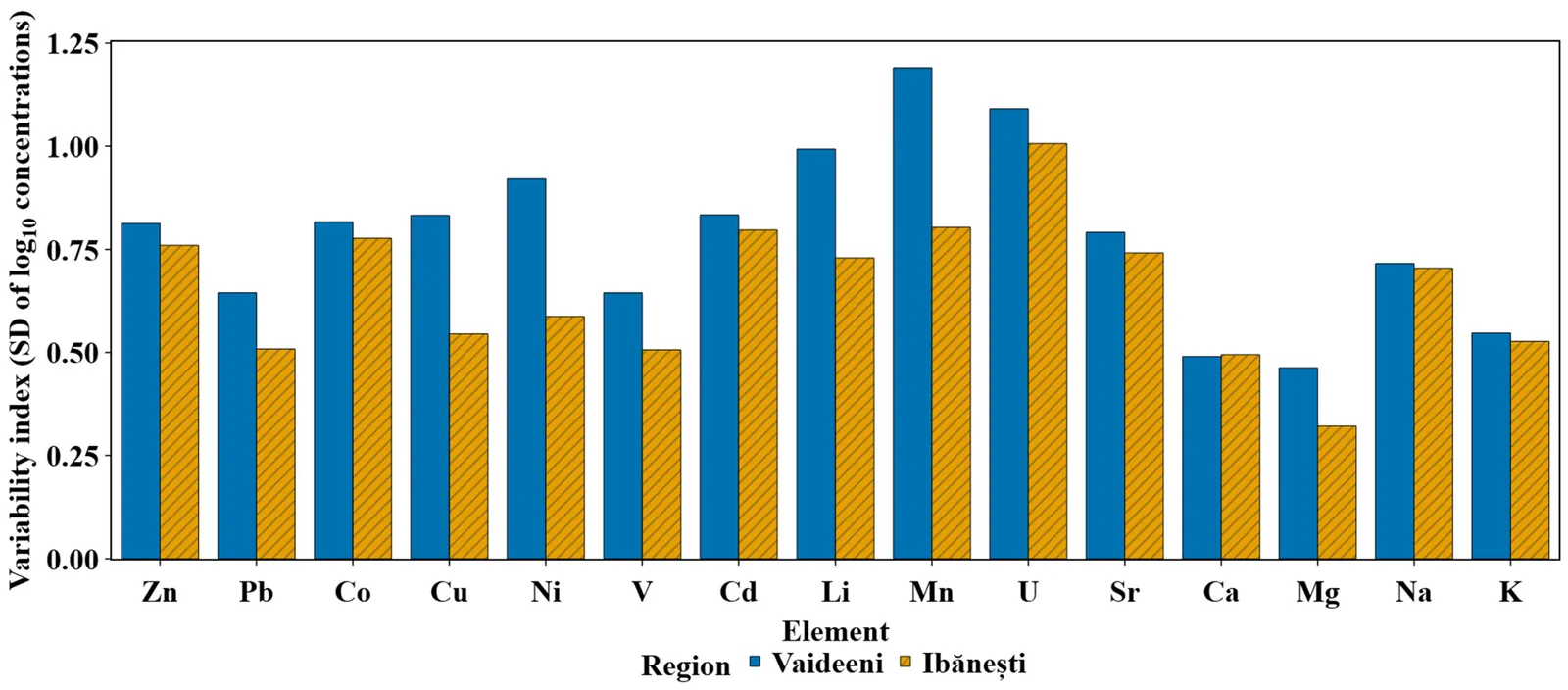
Seasonal variability index of elemental concentrations in dairy products from Vaideeni and Ibănești production systems.

To quantify the temporal stability, a seasonal variability index was calculated as the standard deviation of log-transformed concentrations for each element and region. The resulting values are presented in Figure 7. This approach provided a comparative assessment of the seasonal fluctuations independent of the concentration magnitude and allowed for direct comparison among different elemental groups.

Trace elements displayed the highest seasonal variability, indicating increased sensitivity to changes in the environmental conditions and dairy processing practices. Elements such as Cu, Cd, Ni, and Zn presented the highest variability indices, suggesting that their concentrations were influenced by seasonal changes in feed composition, grazing conditions, and technological processing. Similar observations were reported in various studies investigating the trace element dynamics in milk and dairy products, with trace elements being more responsive to environmental variability than the major constituents [8,48].

On the contrary, the geochemical fingerprint elements presented intermediate variability. Although seasonal fluctuations were evident, elements such as Sr, U, V, and Mn retained clear regional differentiation throughout the year. This finding is important for traceability applications because geographical markers must remain sufficiently stable to preserve the regional identity despite temporal variation. The literature exploiting elemental and isotopic fingerprinting approaches has similar conclusions, postulating that geochemically derived markers often exhibited higher temporal stability than agriculture-influenced trace elements [18,46].

The macroelements showed the lowest variability indices (Figure 7), indicating high temporal stability. Ca and Mg presented limited fluctuation, reflecting their strong physiological regulation and structural association with the casein micelles. Na and K displayed slightly increased variability, but remained considerably more stable than the trace elements. Similar behaviour was also observed in other dairy science studies, with macroelement concentrations showing to be mainly regulated by biological and physicochemical mechanisms rather than by environmental fluctuations [49,50].

An additional finding emerging from Figure 5, Figure 6 and Figure 7 and Figures S1–S4 (Supplementary Material S1) concerns the contrasting behaviour of the two production systems. The seasonal variability was generally higher in Vaideeni than in Ibănești. This difference probably reflects the distinct production practices in the two regions. The artisanal products manufactured in Vaideeni preserve a closer connection to the local environmental conditions and seasonal changes, whereas industrial production in Ibănești introduces increased homogenization through milk blending and technological standardization. Comparable reports for traditional and industrial food systems showed that artisanal products often retained a stronger environmental signature but also exhibited higher temporal variability [43,44].

From a traceability perspective, the combined interpretation of Figure 5, Figure 6 and Figure 7 indicates that seasonal variability did not obscure the underlying regional elemental fingerprint signal. Although the temporal fluctuations contributed to the overall variability, the regional elemental contrasts and processing-related transformations remained the main sources of variation. As a result, the elemental fingerprints identified in this study seem sufficiently robust to support geographical discrimination across different seasons.

Overall, the results indicate that seasonal variability represents a secondary but non-neglectable source of variation within the dairy chain. Trace elements presented the highest temporal responsiveness, geochemical fingerprint elements combined environmental sensitivity with relatively good stability, and macroelements showed high compositional stability. Among the investigated elements, Sr, U, V, and Mn appeared promising for traceability applications because they retained regional differentiation while exhibiting only moderate seasonal variability.

### 3.6. Dietary Exposure and Risk Assessment Associated with Milk Consumption

The dietary exposure assessment was performed using the concentrations measured in individual raw milk samples collected from the Vaideeni and Ibănești production systems. Pb, Cd, and Ni, were of particular interest, considered among the most relevant toxic elements from a food safety perspective. The exposure estimates were calculated for adults (70 kg body weight) and children (20 kg body weight), assuming an average milk consumption of 216.2 g/day.

#### 3.6.1. Concentrations of Potentially Toxic Elements in Milk

Table 4 summarizes the concentrations of Pb, Cd, and Ni in milk samples collected from the two regions.

The mean Pb concentrations observed in both regions (0.0165 and 0.0140 mg L^−1^ for Vaideeni and Ibănești, respectively) remained below the maximum level of 0.020 mg/kg established by current European Union legislation for lead in milk [51]. Nevertheless, the individual samples from both regions approached or slightly exceeded this threshold, indicating localized variability in the environmental exposure sources. Similar observations have been reported in Croatia, Poland, and Serbia, where the Pb concentrations in raw milk occasionally exceeded the European regulatory limits, despite the general low concentrations of other toxic elements [2,8,43]. Cd concentrations were consistently low in all samples, comparable to the values reported for milk produced in other European agricultural regions [9,52]. Ni concentrations exhibited higher variability, mainly for the Ibănești samples, although the overall levels remained within the range commonly reported for bovine milk [48].

#### 3.6.2. Estimated Dietary Exposure

The estimated daily intake (EDI) values normalized to body weight were gathered in Table 5.

As expected, children exhibited substantially increased exposure values compared to adults due to their lower body weight. For all investigated elements, child exposure was about 3.5 times higher than adult exposure under identical consumption scenarios. This finding is consistent with previous dietary exposure studies that identified children as the most vulnerable consumer group with respect to trace element intake through milk and dairy products [9,28].

#### 3.6.3. Comparison with Toxicological Reference Values

To evaluate potential health risks, the calculated exposure values were compared with the toxicological reference values established by EFSA. Pb exposure was compared with the Benchmark Dose Lower Confidence Limit (BMDL01 = 0.5 µg/kg bw/day) associated with developmental neurotoxicity (Table 6) [33]. Cd exposure was compared with the Tolerable Weekly Intake (TWI = 2.5 µg/kg bw/week) [31], whereas Ni exposure was evaluated against the Tolerable Daily Intake (TDI = 13 µg/kg bw/day) (Table 6) [10].

Among the investigated contaminants, Pb represented the highest contribution to the toxicological thresholds. Although none of the calculated exposure values exceeded the EFSA reference level, the contribution reached about 36% of the BMDL01 in children consuming milk from Vaideeni. This observation indicates that Pb remained the most critical toxic element in dairy products, mainly due to its persistence in the environmental matrices and the absence of a recognized safe exposure threshold [33].

Cd exposure represented less than 10% of the TWI even for children, indicating a very low toxicological concern. Similar findings have been reported [9], suggesting limited transfer of Cd from environmental sources into bovine milk due to physiological barriers at the mammary gland level. Ni exposure represented less than 2% of the TDI for all scenarios, indicating that milk only marginally contributed to overall dietary Ni intake [10].

The results obtained in the present study are consistent with previous investigations conducted in European dairy systems. For example, Pb concentrations were reported to range between 0.004 and 0.028 mg kg^−1^ in Croatian milk [2], while Pb was identified as the main contaminant of concern in milk produced in Poland and Serbia [8,43]. On the contrary, Cd and Ni concentrations generally remained well below levels associated with health risks.

Overall, the dietary exposure assessment suggested that the consumption of milk produced in either the traditional Vaideeni system or the industrial Ibănești system did not pose an important health risk with respect to Pb, Cd, or Ni. However, the relatively high contribution of Pb, mainly for children, highlights the importance of continued monitoring for feed and water quality, which represent the primary pathways for trace element transfer within the dairy production chain.

### 3.7. Dietary Exposure and Risk Assessment Associated with Cheese Consumption

In addition to raw milk, dietary exposure to potential toxic elements was evaluated for the main dairy products produced in the investigated production systems. The exposure calculations were performed using the mean concentrations measured for each cheese category and a consumption scenario of 40 g/day for adults (70 kg body weight) and 20 g/day for children (20 kg body weight).

#### 3.7.1. Concentrations of Potentially Toxic Elements in Dairy Products

The concentrations of Pb, Cd, and Ni considerably varied among the dairy products, reflecting the influence of processing, moisture reduction, whey separation, and maturation on elemental redistribution (Table 7).

The concentrations observed in the processed dairy products were generally higher than those observed within raw milk, illustrating that dairy processing may substantially influence elemental distribution. This pattern was observed for Pb and Ni, which showed relatively higher concentrations in several cheese categories, with the highest measured concentrations occurring in hard cheese (0.977 and 0.933 mg/kg, respectively). Their accumulation was consistent with the element-specific retention within the casein-rich curd matrix, combined with the concentration during moisture loss throughout ripening. In contrast, Cd did not exhibit a similar enrichment pattern, its concentrations remaining unchanged or decreasing in several cheese types. This observation suggests that elemental redistribution during cheesemaking may be governed not only by water removal but also by the physicochemical characteristics of the individual elements, including their affinity for milk proteins and their partitioning between the curd and whey. Similar concentration effects have been reported in the literature, where cheese maturation influenced trace element concentrations through a combination of moisture loss, differential partitioning between the curd and whey, and the affinity of individual elements for the protein-rich matrix [9,18,43,52].

Brined hard cheese also presented relatively elevated concentrations of Pb, Cd, and Ni, suggesting that the salting and maturation processes, together with element-specific physicochemical properties, may contribute to the retention and concentration of trace elements. Fresh cheese exhibited intermediate concentrations for all three elements, reflecting its higher moisture content and limited processing compared with the matured cheese varieties.

Among the specialty dairy products, mozzarella presented moderate Pb concentrations while maintaining comparatively low Cd and Ni levels. This behaviour may be associated with its specific manufacturing process, characterized by curd stretching and limited maturation, which may reduce the extent of trace element redistribution and concentration compared with aged cheeses. Similarly, whey cheese contained among the lowest Pb concentrations, consistent with the hypothesis that certain elements preferentially partition into the curd fraction rather than the whey phase during coagulation [9,18,43,52].

The smoked cheese products showed variable elemental profiles. Conventional smoked cheese contained relatively low Cd and Ni concentrations, whereas the smoked cheese with paprika and smoked cheese with pepper had slightly higher Pb and Ni concentrations. The smoked cheese with pepper presented the highest Pb concentration among the specialty cheeses. Although the limited number of samples precludes definitive conclusions, these findings suggest that additional ingredients and smoking treatments may contribute to the modification of elemental composition through a combination of moisture loss during processing, element-specific physicochemical properties, and the introduction of minor exogenous elemental inputs.

Bellows cheese had comparatively low Ni concentrations and moderate Pb levels, indicating that traditional manufacturing and maturation practices did not result in the substantial enrichment observed for the hard cheese. The relative stable elemental profile of bellows cheese may reflect differences in moisture retention and ripening intensity [9,43,52].

Sour cream exhibited the lowest Ni concentration among all investigated products and relative low Cd levels, while Pb remained moderate. Because sour cream is mainly derived from the fat-rich fraction of milk rather than the casein-rich curd, its elemental profile differs from that of cheese products. The lower concentrations observed for Ni and Cd suggest a preferential association of these elements with protein-rich fractions, whereas the absence of a strong concentration effect in sour cream reflects the limited moisture loss occurring during its manufacture [9,43,52].

The observed elemental patterns may reflect the contribution of technological processes applied during dairy manufacture, including coagulation, whey separation, salting, smoking, and maturation, to the final distribution of trace elements. The products undergoing extensive moisture reduction and ripening, mainly hard and brined hard cheeses, exhibited the highest enrichment, consistent with the combined effects of moisture loss and element-specific physicochemical properties influencing the redistribution of individual elements during cheesemaking. In contrast, the high-moisture products such as whey cheese and sour cream generally retained lower concentrations. These findings are consistent with the hypothesis that dairy processing may act as a selective transformation step in which both technological processes and the physicochemical characteristics of individual elements contribute to the modification of the elemental fingerprints inherited from the raw milk.

#### 3.7.2. Estimated Dietary Exposure

Estimated daily intake values calculated for adults and children are presented in Table 8.

The estimated daily intake (EDI) values were consistently higher for children than for adults owing to their lower body weight, which increased the exposure on a body weight basis (Table 8). However, the magnitude of exposure strongly depended on the type of dairy product consumed, reflecting the processing-induced redistribution of trace elements previously observed within the concentration data.

Among all investigated products, hard cheese generated the highest estimated exposure to both Pb and Ni, with EDI values reaching 0.977 and 0.933 μg/kg bw/day for children and 0.558 and 0.533 μg/kg bw/day for adults, respectively. These results directly reflect the elevated concentrations measured in this product and are consistent with the enrichment associated with the prolonged maturation and moisture reduction. Similar concentration phenomena were reported for aged cheeses, where water loss during ripening may increase the concentration of both nutrients and trace contaminants within the casein-rich matrix [9,18,43,44,52].

Brined hard cheese represented the second-largest contributor to dietary exposure, mainly for Ni, which reached EDI values up to 0.267 μg/kg bw/day in children and 0.153 μg/kg bw/day in adults. The combined effects of coagulation, whey removal, salting, and maturation probably may promote the retention of trace elements within the curd fraction, resulting in higher exposure compared with fresh cheese products [18,44].

The products fresh cheese, bellows cheese, and whey cheese presented intermediate to low exposure values. Although fresh cheese and bellows cheese contained measurable concentrations of Pb, Cd, and Ni, the estimated dietary intakes remained below those observed for hard cheese. Whey cheese generated among the lowest Pb exposure values (0.064 μg/kg bw/day for children and 0.036 μg/kg bw/day for adults), supporting the hypothesis that a portion of the investigated elements preferentially associated with the curd phase rather than remaining in the whey fraction during processing.

Among the specialty products, mozzarella had relatively elevated Pb exposure values (0.182 μg/kg bw/day for children and 0.104 μg/kg bw/day for adults), while maintaining moderate Cd and Ni contributions. This pattern suggests that although mozzarella underwent limited maturation, the stretching and concentration of curd solids may still influence elemental retention. The smoked cheese products presented variable exposure profiles, reflecting the differences in formulation and processing. The conventional smoked cheese presented relative low EDI values for Cd and Ni, whereas the smoked cheese with paprika and smoked cheese with pepper presented moderately increased Pb and Ni exposure. The smoked cheese with pepper presented the highest Pb exposure among the specialty products (0.277 μg/kg bw/day for children and 0.158 μg/kg bw/day for adults), although the small number of analysed samples may warrant cautious interpretation.

Sour cream presented the lowest Ni-related dietary exposure from all investigated products, corresponding to EDI values of 0.081 μg/kg bw/day for children and 0.046 μg/kg bw/day for adults. Cd exposure was low, while Pb exposure remained moderate. Because sour cream was mainly derived from the milk fat fraction rather than the protein-rich curd matrix, the lower exposure values are consistent with the hypothesis that Ni and Cd are preferentially associated with the casein-rich fractions and become concentrated during cheese manufacture rather than cream separation.

The exposure assessment indicated that the type and intensity of dairy processing played an important role in determining dietary intake patterns. The products undergoing intensive moisture reduction and maturation, mainly hard and brined hard cheeses, contributed to the highest exposure levels, whereas high-moisture or fat-rich products such as whey cheese and sour cream generated comparatively lower levels. Despite these differences, all estimated exposure values remained well below the corresponding toxicological reference values, indicating that consumption of the investigated dairy products does not pose an important health risk for either adults or children under the considered consumption scenarios.

The dietary exposure assessment was designed as a screening-level evaluation of potential non-carcinogenic risk. Estimated daily intake (EDI) values were calculated for Pb, Cd, and Ni and subsequently compared with the corresponding health-based guidance values to determine whether the measured concentrations approached levels of toxicological concern. Accordingly, the assessment provided an initial risk characterization rather than a comprehensive quantitative health risk assessment.

#### 3.7.3. Comparison with Toxicological Reference Values

To assess the potential health risks, the exposure values were compared with the EFSA Benchmark Dose Lower Confidence Limit for Pb (BMDL01 = 0.5 μg/kg bw/day), the Tolerable Weekly Intake for Cd (TWI = 2.5 μg/kg bw/week), and the Tolerable Daily Intake for Ni (TDI = 13 μg/kg bw/day) [10,33]. These reference values provided a framework for evaluating the significance of dietary exposure and identifying potential elements of concern.

Among the investigated contaminants, Pb was the most relevant contributor to dietary exposure. The highest estimated daily intake was associated with the hard cheese, reaching 0.977 μg/kg bw/day for children and 0.558 μg/kg bw/day for adults (Table 8). These values exceeded the EFSA BMDL01, corresponding to about 195% and 112% of the reference value, respectively. Nevertheless, it should be stated that the BMDL01 is not a health-based guidance value defining a threshold for adverse effects but rather a reference point used for risk characterization. As a result, the observed exceedance may not directly imply the occurrence of adverse health effects but may highlight the importance of considering Pb exposure from cheese within the context of total dietary intake. The elevated Pb exposure associated with hard cheese is consistent with the combined effects of moisture loss during ripening and element-specific physicochemical properties, including preferential retention within the casein-rich curd matrix and curd–whey partitioning. These concentration effects should not be interpreted as a universal behaviour for all elements because the elemental redistribution during cheesemaking depends on the physicochemical characteristics of each element, including their affinity for the protein-rich curd matrix and partitioning between the curd and whey. Intermediate Pb exposure values were observed for mozzarella, smoked cheese products, brined hard cheese, bellows cheese, and sour cream. Among the specialty cheeses, the smoked cheese with pepper presented the highest Pb-related exposure (0.277 μg/kg bw/day for children), while the whey cheese exhibited the lowest contribution (0.064 μg/kg bw/day for children). These differences further suggest the influence of manufacturing practices, maturation intensity, and product composition on the final contaminant burden.

On the contrary, Cd exposure remained very low across all investigated products. Even for the brined hard cheese, which presented the highest Cd concentration, the estimated weekly intake represented only a small fraction of the EFSA TWI. In a similar manner, Ni exposure remained below the established TDI for all dairy products. The highest Ni-related exposure was observed for the hard cheese, corresponding to less than 8% of the TDI, indicating a low toxicological concern under the considered consumption scenarios.

The exposure patterns closely mirrored the concentration profiles observed within the dairy products, indicating that processing-induced enrichment directly influenced dietary intake. The products subjected to extensive maturation and moisture reduction, in particular hard and brined hard cheeses, generated higher exposure values than fresh, whey-based, or fat-rich products such as sour cream. Similar studies reported that, for traditional and artisanal cheeses, coagulation, whey separation, and ripening were identified as the key factors governing the concentration of trace elements during processing [8,9,18,43,44,48,52].

Overall, risk characterization indicates that cheese consumption is unlikely to represent an important source of Cd or Ni exposure for either adults or children. However, the relatively high Pb exposure associated with certain matured cheeses highlights the importance of continued monitoring for Pb contamination throughout the dairy production chain. These findings suggest that dairy processing not only redistributs the elemental signatures but may also increase contaminant concentrations in a specific product category, thereby influencing the final dietary exposure of consumers.

## 4. Conclusions

This study investigated the propagation, transformation, and traceability of the elemental fingerprints along the feed–animal–milk–products chain in two contrasting Romanian dairy production systems, represented by the traditional Vaideeni region and the industrial Ibănești region. The results evidence that the distinct regional elemental signatures were already established at the environmental input level, with the water mainly reflecting the hydrogeochemical characteristics and the feed reflecting the agricultural management practices and biomass sourcing.

Although pronounced regional differences were observed within the water and feed, the transfer of the elemental signatures into the milk was moderated by the physiological processes. The milk matrix acted as a biological filter, attenuating the environmental contrasts while preserving measurable differences for the selected elements. Among the investigated elements, Li, U, Sr, Mn, and Cu presented the highest discriminatory potential, indicating that these geochemical and trace element markers retained valuable information for differentiating the two investigated dairy production systems despite biological regulation.

The comparison of the elemental fingerprints across the water, feed, and milk was consistent with a propagation–attenuation pattern for elemental transfer within the dairy chain. The correlation analysis evidenced that environmental availability alone could not fully explain the milk composition, highlighting the importance of selective absorption, metabolic regulation, and mammary gland transfer mechanisms. These findings support the hypothesis that milk represents an integrated and biologically modified expression of environmental exposure rather than a direct reflection of the environmental concentrations.

Dairy processing introduced an additional level of complexity through the selective redistribution of the elements among the product fractions. The trace elements presented the highest sensitivity to technological processing, whereas the macroelements remained comparatively stable. The geochemical fingerprint elements kept a substantial part of their regional information despite the processing-induced modification, demonstrating their potential suitability as robust markers for dairy product authentication and geographical differentiation within the investigated production systems.

Seasonal variability analysis further highlighted that temporal fluctuations did not obscure the underlying geographical signal. While the trace elements had increased seasonal variability, the geochemical fingerprint elements such as Sr, U, V, and Mn combined regional contrasts with relatively good temporal stability, reinforcing their value for provenance studies and supporting future geographical traceability applications following broader geographical validation.

The dietary exposure assessment indicated that the estimated daily and weekly intakes of the investigated elements through milk consumption remained below the toxicological reference values for both adults and children. Overall, the analysed samples of milk and dairy products did not pose an important health risk under the considered consumption scenarios. However, the occurrence of elevated lead concentrations in some samples evidenced the importance of continued monitoring of environmental inputs and dairy products to guarantee consumer safety and compliance with the regulatory requirements.

Overall, the results suggest that multielement fingerprinting may provide complementary information for investigating elemental traceability, food authenticity, and regional elemental patterns within the two investigated dairy production systems. The integration of biological, environmental, seasonal, and technological information may provide a comprehensive framework to understand elemental transfer mechanisms and identify candidate markers for geographical differentiation. Nevertheless, the present study was designed to compare two contrasting production systems rather than to develop or validate a predictive geographical authentication model. As a result, the identified elemental fingerprints should be regarded as promising indicators within the investigated systems rather than universal geographical markers. Future investigation should include broader geographical coverage, larger datasets, isotopic markers, and validated multivariate classification approaches (e.g., PCA, PLS-DA, Random Forest, or other machine-learning models) before elemental fingerprints can be reliably applied as authentication tools for dairy products across wider geographical regions.

## Figures and Tables

**Table 1:** Regional arithmetic mean concentrations of elements in water and feed samples, used as reference values to calculate the corresponding Δlog_10_ (Ibănești–Vaideeni).

Element	Water Vaideeni (mg/L)	Water Ibănești (mg/L)	Δlog10 Water	Feed Vaideeni (mg/kg)	Feed Ibănești (mg/kg)	Δlog10 Feed
Zn	0.021	0.009	−0.368	26.502	13.215	−0.302
Pb	0.002	0.001	−0.301	0.154	0.168	0.040
Co	0.0004	0.0008	0.301	0.040	0.023	−0.236
Cu	0.008	0.011	0.138	4.228	1.416	−0.475
Ni	0.003	0.004	0.125	1.294	0.305	−0.628
V	0.0004	0.008	1.301	0.066	0.043	−0.184
Cd	0.0003	0.0006	0.301	0.059	0.028	−0.331
Li	0.025	0.011	−0.357	0.038	0.035	−0.032
Mn	0.002	0.008	0.602	51.920	13.027	−0.601
U	0.0003	0.002	0.824	0.034	0.062	0.256
Sr	0.072	0.295	0.612	5.422	2.250	−0.382
Ca	25.570	72.070	0.450	650.179	199.824	−0.512
Mg	11.400	26.580	0.368	2166.350	746.447	−0.463
Na	56.530	154.000	0.435	221.676	79.118	−0.447
K	13.770	54.140	0.595	8390.079	2230.977	−0.575

**Table 2:** Regional arithmetic mean concentrations of elements in milk samples used as reference values to calculate the corresponding Δlog_10_ (Ibănești–Vaideeni).

Element	Milk Vaideeni (mg/L)	Milk Ibănești (mg/L)	Δlog_10_ Milk (Ibănești–Vaideeni)
Zn	2.2413	2.2058	−0.0069
Pb	0.0165	0.0140	−0.0724
Co	0.0020	0.0030	0.1794
Cu	0.0682	0.0150	−0.6574
Ni	0.0152	0.0198	0.1150
V	0.0078	0.0050	−0.1914
Cd	0.0022	0.0028	0.1000
Li	0.0122	0.0026	−0.6697
Mn	0.0207	0.0122	−0.2277
U	0.2317	0.0061	−1.5813
Sr	0.6023	0.2462	−0.3884
Ca	556.9231	503.4688	−0.0438
Mg	123.3471	121.8719	−0.0052
Na	430.4038	417.9688	−0.0127
K	1304.3269	1034.4072	−0.1007

**Table 3:** Spearman and Pearson correlations between environmental inputs and milk elemental contrasts.

Comparison	Spearman Correlation (ρ)	*p*-Value	Pearson Correlation (r)	*p*-Value
Water vs. Milk	−0.35	0.196	−0.19	0.493
Feed vs. Milk	−0.26	0.348	−0.60	0.018

**Table 4:** Concentrations of potentially toxic elements in milk samples (mg/L).

Region	Element	Mean Values ± SD (mg/L)	Range (mg/L)
Vaideeni	Pb	0.0165 ± 0.0063	0.0080–0.0270
Ibănești	Pb	0.0140 ± 0.0080	0.0040–0.0210
Vaideeni	Cd	0.0022 ± 0.0010	0.0002–0.0030
Ibănești	Cd	0.0028 ± 0.0010	0.0020–0.0040
Vaideeni	Ni	0.0152 ± 0.0104	0.0020–0.0290
Ibănești	Ni	0.0198 ± 0.0154	0.0020–0.0380

**Table 5:** Estimated daily intake (EDI) of potential toxic elements through milk consumption.

Region	Element	Adult EDI (µg/kg bw/Day)	Child EDI (µg/kg bw/Day)
Vaideeni	Pb	0.0511	0.1788
Ibănești	Pb	0.0432	0.1513
Vaideeni	Cd	0.0067	0.0236
Ibănești	Cd	0.0085	0.0297
Vaideeni	Ni	0.0468	0.1638
Ibănești	Ni	0.0610	0.2135

**Table 6:** Contribution of milk consumption to toxicological reference values (%).

Region	Element	Adult (%)	Child (%)	Reference Value
Vaideeni	Pb	10.2	35.8	BMDL01
Ibănești	Pb	8.6	30.3	BMDL01
Vaideeni	Cd	1.9	6.6	TWI
Ibănești	Cd	2.4	8.3	TWI
Vaideeni	Ni	0.36	1.26	TDI
Ibănești	Ni	0.47	1.64	TDI

**Table 7:** Concentrations of Pb, Cd, and Ni in dairy products (mg/kg, fresh weight basis).

Product	*n*	Pb (mg/kg)	Cd (mg/kg)	Ni (mg/kg)
Fresh cheese	18	0.086	0.018	0.126
Brined hard cheese	13	0.120	0.024	0.267
Hard cheese	7	0.977	0.007	0.933
Bellows cheese	4	0.091	0.017	0.095
Whey cheese	4	0.064	0.017	0.113
Mozzarella	4	0.182	0.011	0.124
Smoked cheese	2	0.123	0.005	0.100
Smoked cheese with paprika	2	0.124	0.010	0.144
Smoked cheese with pepper	1	0.277	0.0001	0.136
Sour cream	4	0.099	0.012	0.081

**Table 8:** Estimated daily intake (EDI) associated with cheese consumption (μg/kg bw/day).

Product	Pb (Adult)	Pb (Child)	Cd (Adult)	Cd (Child)	Ni (Adult)	Ni (Child)
Fresh cheese	0.049	0.086	0.010	0.018	0.072	0.126
Brined hard cheese	0.069	0.120	0.014	0.024	0.153	0.267
Hard cheese	0.558	0.977	0.004	0.007	0.533	0.933
Bellows cheese	0.052	0.091	0.010	0.017	0.054	0.095
Whey cheese	0.036	0.064	0.010	0.017	0.065	0.113
Mozzarella	0.104	0.182	0.006	0.011	0.071	0.124
Smoked cheese	0.070	0.123	0.003	0.005	0.057	0.100
Smoked cheese with paprika	0.071	0.124	0.005	0.010	0.082	0.144
Smoked cheese with pepper	0.158	0.277	0.0001	0.0001	0.078	0.136
Sour cream	0.057	0.099	0.007	0.012	0.046	0.081

## Data Availability

The original contributions presented in this study are included in the article/Supplementary Materials. Further inquiries can be directed to the corresponding authors.

## Supplementary Materials

The following supporting information can be downloaded at: https://www.mdpi.com/article/10.3390/foods15162844/s1, Supplementary Material S1: Table S1. Mineralization program (microwave digestion of aqueous samples) used for water sample preparation; Table S2. Mineralization program (microwave digestion of straw) used for animal feed sample preparation); Table S3. Mineralization program (microwave digestion of wheat) used for animal feed sample preparation); Table S4. Mineralization program (microwave digestion of milk (whole liquid)) used for milk sample preparation); Table S5. Mineralization program (microwave digestion of cheese) used for cheese sample preparation; Table S6. ICP-OES instrumental parameters for multielement determination; Table S7. Combined analytical validation parameters for water, feed, milk and cheese; Table S8. Flame Atomic Absorption Spectroscopy (FAAS) instrumental parameters; Table S9. Combined analytical validation parameters for water, feed, milk and cheese; Figure S1. Seasonal evolution of macroelements (Ca, Mg, Na, and K) in milk and dairy products from the Vaideeni region; Figure S2. Seasonal evolution of macroelements (Ca, Mg, Na, and K) in milk and dairy products from the Ibănești region; Figure S3. Seasonal evolution of trace elements in Vaideeni region; Figure S4. Seasonal evolution of trace elements in Ibănești region. Supplementary Material S2: Sample distribution; element concentrations.
