# Supplementary material for: Traceability and Health Risk Assessment in the Feed–Animal–Milk Chain: A Comparative Evaluation of Traditional and Industrial Dairy Systems in Romania
Source: Foods. 2026 Aug 14;15(16):2844. doi: 10.3390/foods15162844 (PMC13512816; doi:10.3390/foods15162844)
Supplement: Supplementary file 1 [file foods-15-02844-s001.zip › Supplementary material S1.pdf]

- *Supplementary Material S1 –*

Traceability and Health Risk Assessment in the Feed-Animal-Milk Chain: Comparative Evaluation of Traditional and Industrial Dairy Systems in Romania

Ionela Ramona Zgavarogea [1], Nadia Paun [1,*], Claudia Sandru[1], Violeta-Carolina Niculescu [1,*], Ana Maria Nasture [1,2], Marius-Gheorghe Miricioiu[1]

[1]National Research and Development Institute for Cryogenic and Isotopic Technologies—ICSI Ramnicu Vâlcea, 4th Uzinei Street, P.O. Box Raureni 7, 240050 Ramnicu Valcea, Romania; ramona.zgavarogea@icsi.ro (I.R.Z.); ana.nasture@icsi.ro (A.M.N.)

[2]DAIC SYSTEM S.R.L., 58 Fantanii Street, 240301 Ramnicu Valcea, Romania

*****Correspondence: nadia.paun@icsi.ro (N.P.); violeta.niculescu@icsi.ro (V.-C.N.)

Table S1. Mineralization program (microwave digestion of aqueous samples) used for water sample preparation

Parameter	Microwaves Power (W)	Ramp Time (min)	Pressure (psi)	Holding Temperature (°C)	Holding Time (min)
Value	900−1800	10	800	170	10

Table S2. Mineralization program (microwave digestion of straw) used for animal feed sample preparation)

Parameter	Microwaves Power (W)	Ramp Time (min)	Pressure (psi)	Holding Temperature (°C)	Holding Time (min)
Value	900−1800	15	800	200	15

Table S3. Mineralization program (microwave digestion of wheat) used for animal feed sample preparation)

Parameter	Microwaves Power (W)	Ramp Time (min)	Pressure (psi)	Holding Temperature (°C)	Holding Time (min)
Value	900−1800	15	800	200	15

Table S4. Mineralization program (microwave digestion of milk (whole liquid)) used for milk sample preparation)

Parameter	Microwaves Power (W)	Ramp Time (min)	Pressure (psi)	Holding Temperature (°C)	Holding Time (min)
Value	900−1800	15	800	200	15

Table S5. Mineralization program (microwave digestion of cheese) used for cheese sample preparation

Parameter	Microwaves Power (W)	Ramp Time (min)	Pressure (psi)	Holding Temperature (°C)	Holding Time (min)
Value	900−1800	20	800	210	15

Table S6. ICP-OES instrumental parameters for multielement determination

Parameters /Element	Wavelength (nm)	Gas type	Plasma view	Calibration range
**Zn**	213.856	Ar	Axial	
**Pb**	220.353	Ar	Axial	
**Co**	228.615	Ar	Axial	
**Cu**	327.396	Ar	Axial	
**Ni**	221.648	Ar	Axial	
**V**	292.401	Ar	Axial	5.0 – 100.0 µg/L
**Cd**	226.502	Ar	Axial	
**Li**	670.791	Ar	Radial	
**Mn**	257.610	Ar	Axial	
**U**	385.957	**Ar**	Axial	
**Sr**	421,552	Ar	Radial	

Table S7. Combined analytical validation parameters for water, feed, milk and cheese

Matrix	Element	R^2	Standards	Matrix Match	LOD (µg/L)	LOQ (µg/L)	Precision (%RSDr)	Uncertainty (%)	Recovery (%)	Spectral Interference Correction
	Zn	0.99960	5	Yes	0.0084	0.0249	2.893	9	101.213	Corrected by spectral separation and background correction
	Pb	0.99996	5	Yes	0.0254	0.0763	3.3145	13	93.398	No correction required
	Co	0.99997	5	Yes	0.00104	0.0031	4.049	13	95.056	No correction required
	Cu	0.99992	5	Yes	0.00349	0.0104	1.678	10	90.242	No correction required
	Ni	0.99986	5	Yes	0.00438	0.0131	5.2909	11	93.144	No correction required
Water	V	0.99997	5	Yes	0.00668	0.0201	2.2376	9	96.5477	No correction required
	Cd	0.99997	5	Yes	0.00854	0.0108	6.069	3	90.932	No correction required
	Li	0.99995	5	Yes	0.00526	0.01579	1.774	8	105.900	No correction required
	Mn	0.99997	5	Yes	0.00176	0.00529	4.5702	9	99.235	No correction required
	U	0.99991	5	Yes	2.518	7.555	4.1371	9	93.552	Background corrected by background shift; evaluated with blank matrix solution
	Sr	0.99998	5	Yes	0.01183	0.03548	2.2214	12	91.550	No correction required
	Zn	0.99970	5	Yes	0.5916	1.775	2.2909	10	97.024	No correction required

	Pb	0.99984	5	Yes	6.210	18.63	7.9443	19	84.766	Corrected by spectral separation and background correction
	Co	0.99960	5	Yes	1.213	3.639	9.3705	20	97.412	No correction required
	Cu	0.99979	5	Yes	0.9851	2.955	2.8676	11	99.133	No correction required
Feed (cereals)	Ni	0.99983	5	Yes	0.8170	1.685	6.9264	21	97.975	Corrected by spectral separation and background correction
	V	0.99983	5	Yes	0.4155	1.246	2.7594	17	96.930	No correction required
	Cd	0.99974	5	Yes	0.2442	0.7326	8.4565	20	105.776	No correction required
	Li	0.99978	5	Yes	0.8170	2.451	4.1301	9	99.876	Corrected by spectral separation and background correction
	Mn	0.99946	5	Yes	0.07897	2.451	1.5811	12	104.99	No correction required
	U	0.99985	5	Yes	3.4410	7.32	5.8378	8	97.433	Background corrected by background shift; evaluated with blank matrix solution
	Sr	0.99981	5	Yes	0.02383	0.0715	1.5810	18	104.19	No correction required
	Zn	0.99591	5	Yes	0.08207	0.2462	2.893	9	98.213	Corrected by spectral separation and background correction
	Pb	0.99960	5	Yes	1.649	4.946	6.600	15	87.931	No correction required
	Co	0.99977	5	Yes	0.1192	0.3575	6.049	14	96.056	No correction required
	Cu	0.99959	5	Yes	0.2378	0.7135	9.758	12	97.016	No correction required
	Ni	0.99937	5	Yes	0.2288	0.6863	6.855	18	99.189	No correction required
Milk and Cheese	V	0.99976	5	Yes	0.1230	0.3691	2.2376	13	97.547	No correction required
	Cd	0.99958	5	Yes	0.05663	0.1699	9.069	7	101.510	No correction required
	Li	0.99993	5	Yes	0.5512	1.654	5.902	8	102.439	Corrected by spectral separation and background correction
	Mn	0.99985	5	Yes	0.01998	0.0599	4.5702	10	102.235	No correction required
	U	0.99942	5	Yes	2.1120	4.3179	6.449	10	97.021	Background corrected by background shift; evaluated with blank matrix solution
	Sr	0.99989	5	Yes	0.01183	0.03548	1.2214	16	95.550	No correction required

Table S8. Flame Atomic Absorption Spectroscopy (FAAS) instrumental parameters

Parameters/Element	Wavelength (nm)	Gas type	Slit width (nm)	Lamp current (mA)	Calibration rang (mg/L)
**Ca**	422.7	Air - Acetylene	1.2	3.0	0.50 – 5.00
**Mg**	285.2	Air - Acetylene	1.2	2.0	0.05 – 0.50
**Na**	589.0	Air - Acetylene	0.8	3.0	0.10 – 1.00
**K**	766.5	Air - Acetylene	0.8	4.0	0.10 – 1.00

Table S9. Combined analytical validation parameters for water, feed, milk and cheese

Matrix	Element	R^2	Standards	Matrix Match	LOD (mg/L)	LOQ (mg/L)	Precision (%RSDr)	Uncertainty (%)	Recovery (%)	Background Correction
Water	Ca	0.9981	6	Yes	0.150	0.500	2.04	19	105	No correction required
	Mg	0.9978	6	Yes	0.015	0.050	1.12	15	98	No correction required
	Na	0.9974	5	Yes	0.030	0.100	0.95	23	108	No correction required
	K	0.9979	5	Yes	0.030	0.100	2.74	11	104	No correction required
Feed (cereals)	Ca	0.9981	6	Yes	0.150	0.500	2.17	8	101	No correction required
	Mg	0.9978	6	Yes	0.010	0.050	1.91	6	104	No correction required
	Na	0.9974	5	Yes	0.030	0.100	2.33	10	109	No correction required
	K	0.9979	5	Yes	0.030	0.100	2.67	15	108	No correction required
Milk and cheese	Ca	0.9981	6	Yes	0.150	0.500	2.83	9	101	No correction required
	Mg	0.9978	6	Yes	0.015	0.050	1.96	13	101	No correction required
	Na	0.9974	5	Yes	0.030	0.100	3.87	9	103	No correction required
	K	0.9979	5	Yes	0.030	0.100	4.64	13	106	No correction required

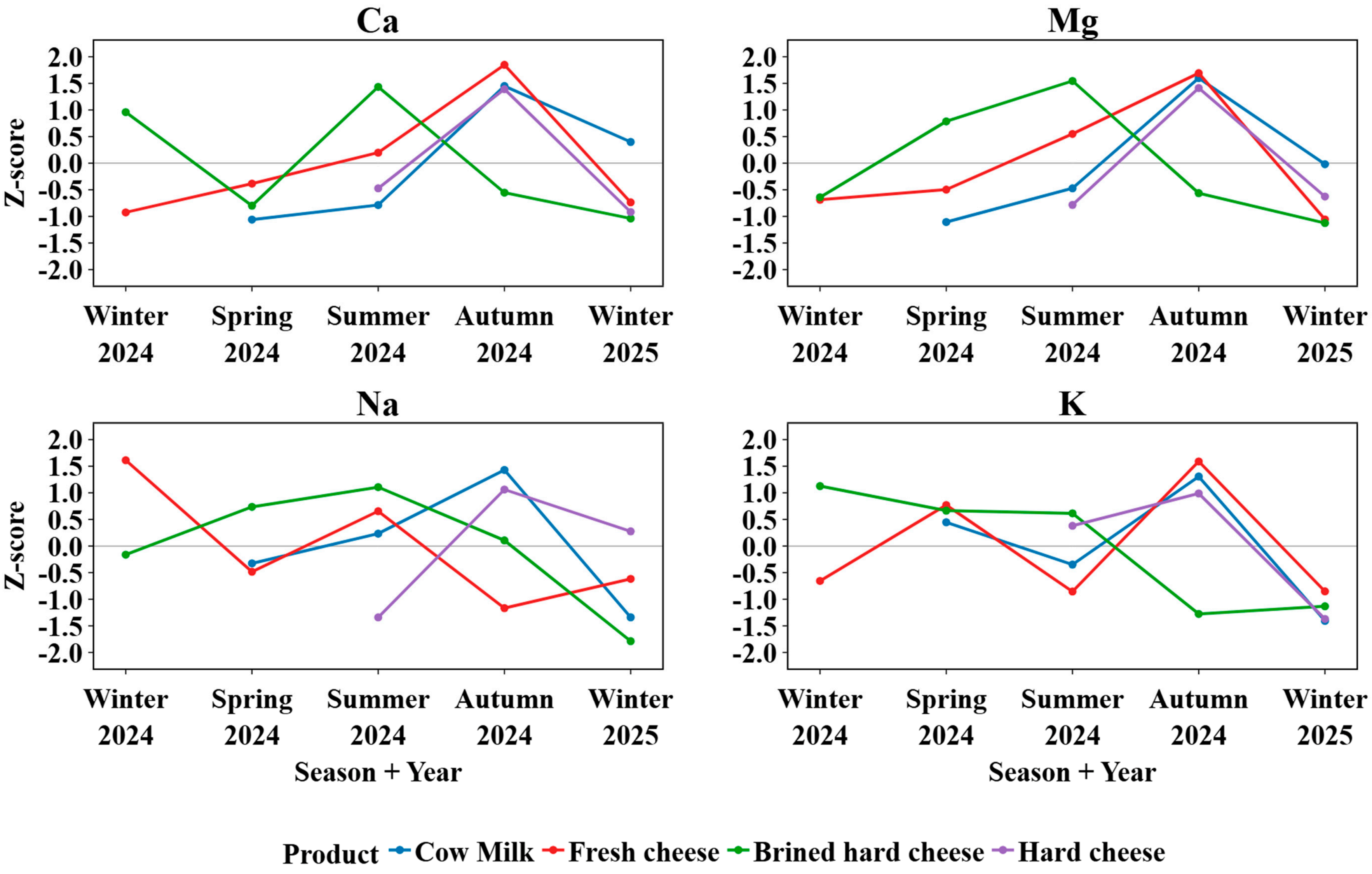

Figure S1. Seasonal evolution of macroelements (Ca, Mg, Na, and K) in milk and dairy products from the Vaideeni region

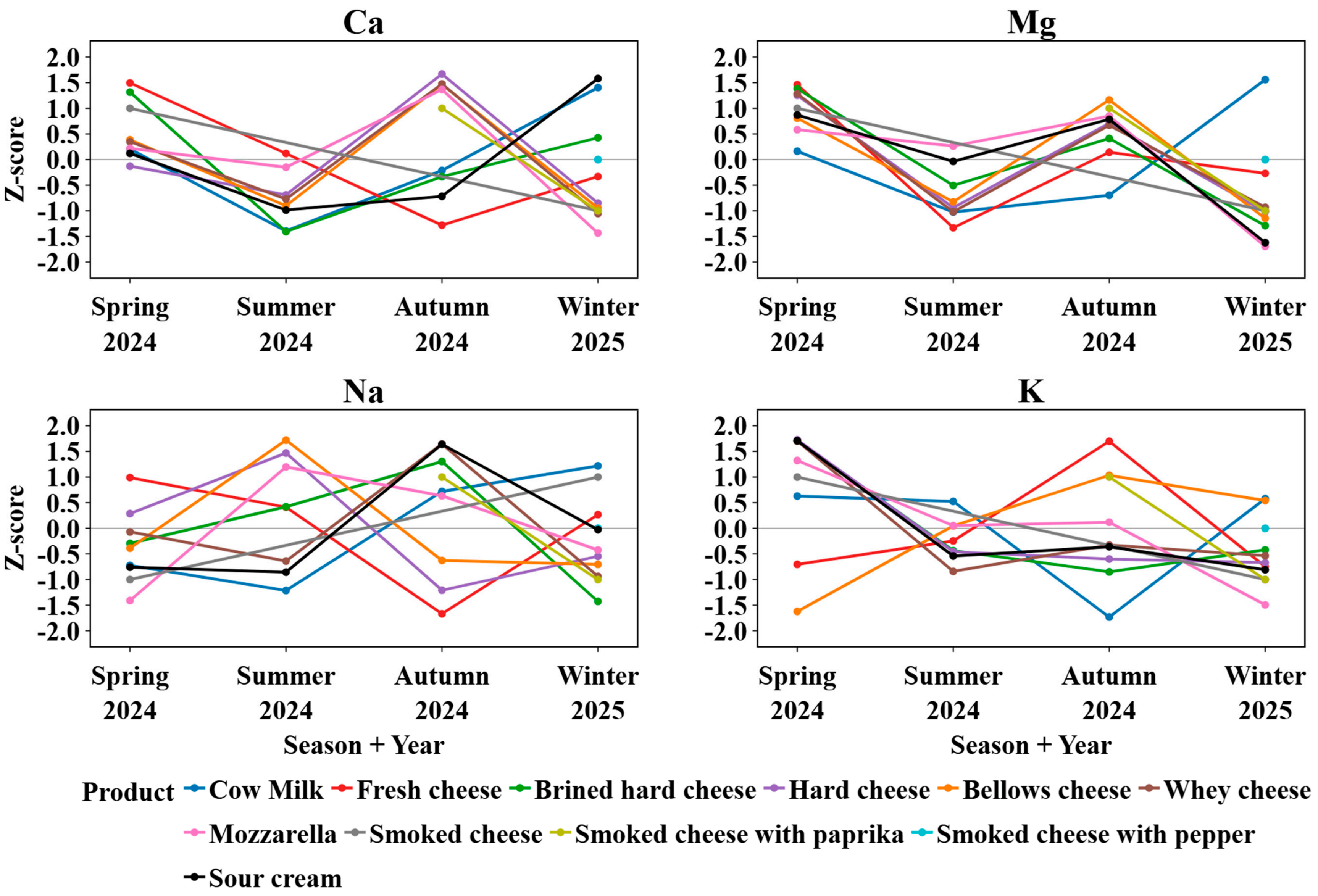

Figure S2. Seasonal evolution of macroelements (Ca, Mg, Na, and K) in milk and dairy products from the Ibănești region

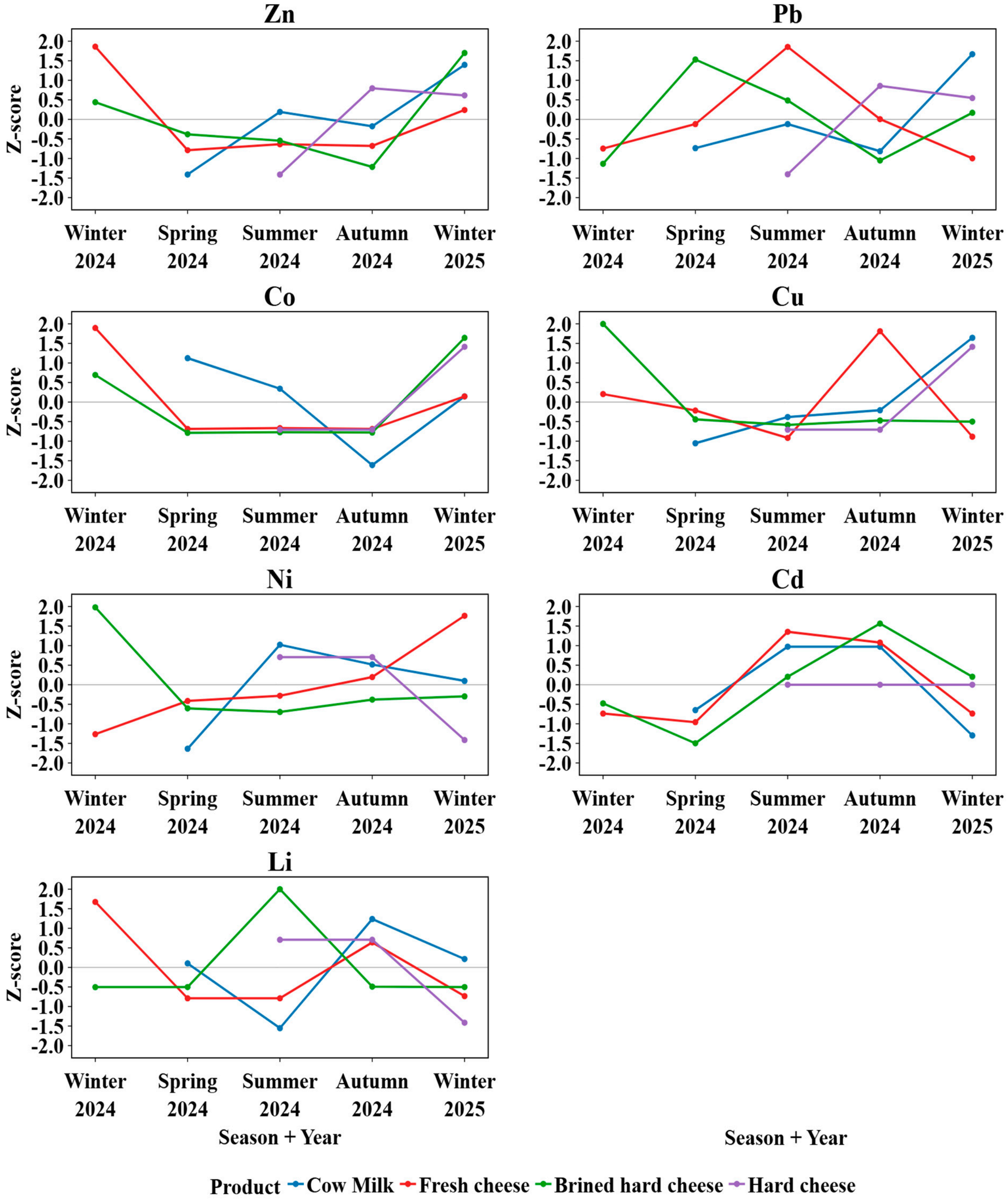

Figure S3. Seasonal evolution of trace elements in Vaideeni region

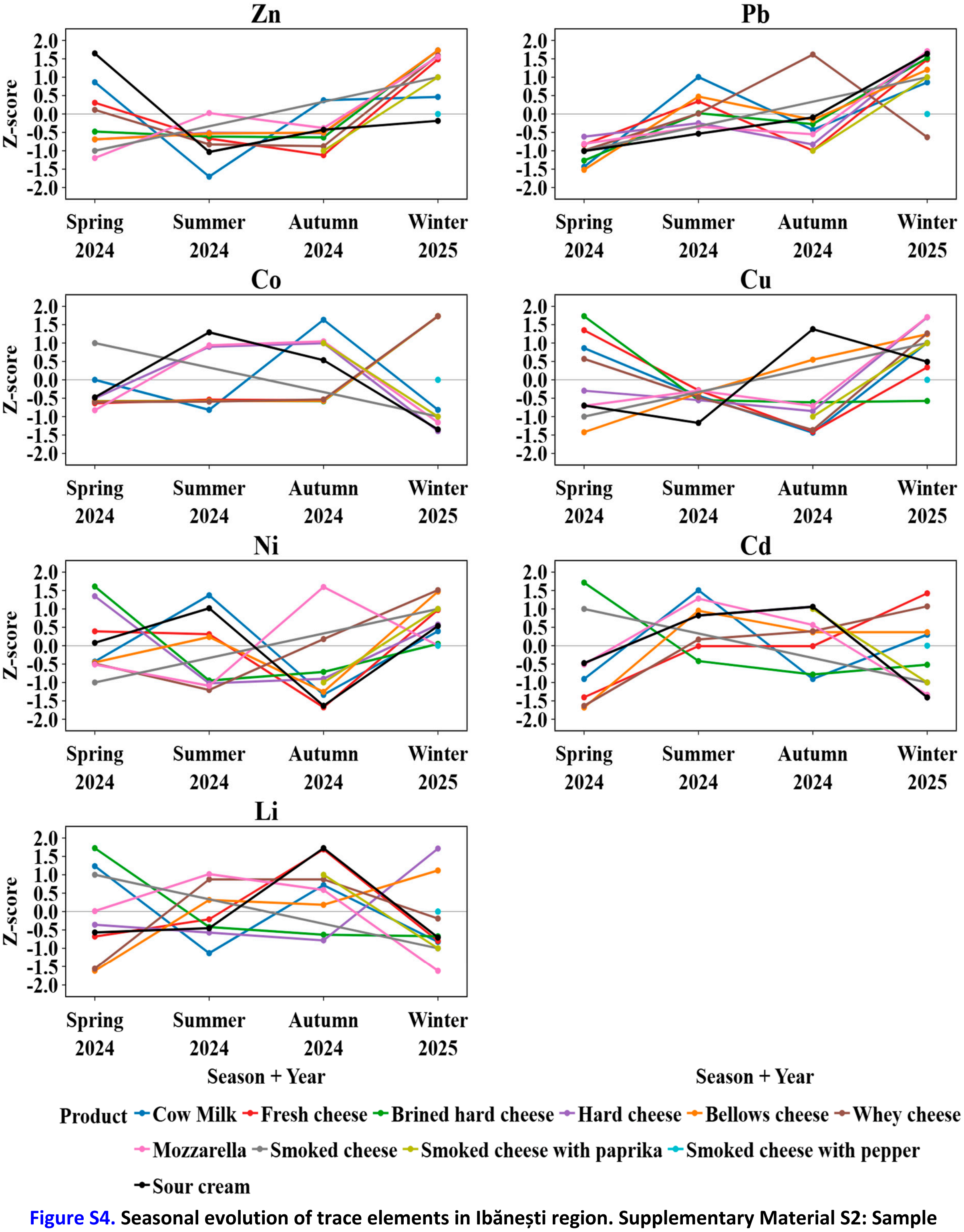

Figure S4. Seasonal evolution of trace elements in Ibănești region. Supplementary Material S2: Sample distribution; element concentrations
